# Supplementary material for: Toward understanding the role of visual hierarchy in flicker-induced time dilation: A preregistered study
Source: PLoS One. 2026 Oct 13;21(10):e0337307. doi: 10.1371/journal.pone.0337307 (PMC13660902; doi:10.1371/journal.pone.0337307)
Supplement: Supplementary file 1 [file pone.0337307.s001.docx]

**Movie S1. An exemplary semantic flicker sequence.**

**Movie S2. An exemplary scramble sequence. This sequence is derived from the same image shown in Movie S1**

**Movie S3. An exemplary luminance flicker sequence. This sequence is derived from the same image shown in Movie S1.**

**Movie S4. An exemplary combined flicker sequence. This sequence is derived from the same image shown in Movie S1.**

# Analysis of ITPC at harmonic frequencies

The 2-Hz ITPC hypothesis-driven analysis procedure was also applied to the harmonic frequencies in a follow-up examination. Therefore, ITPC values from the ROI channels at each harmonic frequency were averaged over the window of 1 to 2 s relative to the sequence onset. Contrasting the ITPC values across conditions revealed that at 4 Hz (Fig S1), all flickering conditions resulted in significant phase clustering relative to the static baseline condition (for luminance, t (18) = -4.59, *p* = 0.002, 95% CI [-0.213, -0.038], Cohen’s *d* = -0.91; for semantic, t (18) = -6.12, *p* < 0.001, 95% CI [-0.361,- 0.113], Cohen’s *d* = -1.71; and for combined flicker conditions, t (18) = -5.85, *p* < 0.001, 95% CI [-0.493, -0.144], Cohen’s *d* = -2.30). No significant difference in ITPC was found between static and scramble conditions (t (18) = -1.71, *p* = 1, 95% CI [-0.164, 0.049], Cohen’s *d* = -0.41). Moreover, ITPC in luminance and scramble conditions were comparable (t (18) = -1.94, *p* = 0.67, 95% CI [-0.181, 0.043], Cohen’s *d* = -0.49). This is in line with exploratory analysis results revealing there was no significant entrainment at 4 Hz for the luminance flickers (Fig 5). In the same vein, ITPC in semantic (t (18) = -4.47, *p* = 0.003, 95% CI [-0.307, -0.051], Cohen’s *d* = -1.29) and combined flicker (t (18) = -5.55, *p* < 0.001, 95% CI [-0.412, -0.110], Cohen’s *d* = -1.89) conditions was significantly higher than the scramble condition, paralleling the exploratory analysis results. The ITPC was comparable between semantic flickers and luminance (t (18) = -2.69, *p* = 0.15, 95% CI [-0.243, 0.020], Cohen’s *d* = -0.80) and combined flicker (t (18) = -1.81, *p* = 0.86, 95% CI [-0.226, 0.062], Cohen’s *d* = -0.59) conditions. Lastly, the ITPC in combined condition was significantly higher than luminance condition (t (18) = -3.55, *p* = 0.02, 95% CI [-0.366, -0.019], Cohen’s *d* = -1.39).

At 6 Hz (Fig S1), ITPC in static condition was not significantly different from scramble (t (18) = 2.15, *p* = 0.44, 95% CI [-0.026, 0.136], Cohen’s *d* = 0.45) and semantic conditions (t (18) = -2.19, *p* = 0.41, 95% CI [-0.177, 0.032], Cohen’s *d* = -0.60), indicating lack of entrainment at this frequency for the sematic flicker condition. The ITPC in luminance (t (18) = -3.46, *p* = 0.02, 95% CI [-0.232, -0.001], Cohen’s *d* = -1.01) and combined flicker (t (18) = -6.42, *p* < 0.001, 95% CI [-0.376, -0.126], Cohen’s *d* = -2.08) conditions were significantly higher than the baseline static condition. The ITPC in scramble condition was significantly lower than all flickering conditions (for luminance, t (18) = -6.75, *p* < 0.001, 95% CI [-0.259, -0.092], Cohen’s *d* = -1.46; for semantic, t (18) = -4.9, *p* < 0.001, 95% CI [-0.210, -0.044], Cohen’s *d* = -1.05; and for combined flicker conditions, t (18) = -7.03, *p* < 0.001, 95% CI [-0.445, -0.166], Cohen’s *d* = -2.54). ITPC in luminance flicker conditions was comparable to semantic (t(18) = 1.5, *p* = 1, 95% CI [-0.055, 0.152], Cohen’s *d* = 0.41) and combined flickers (t (18) = -3.06, *p* = 0.06, 95% CI [-0.265, 0.005], Cohen’s *d* = -1.08). Lastly, ITPC in combined flicker condition was higher than the semantic flicker condition (t (18) = -4.63, *p* = 0.002, 95% CI [-0.301, -0.055], Cohen’s *d* = -1.48).

Moreover, we assessed the correlation between each of the harmonic frequencies and PSEs across subjects in all conditions. No significant correlation was found (Fig S2).

**Fig S1. Analysis of ITPC values at harmonic frequencies averaged over ROI electrodes and the time window of 1 to 2 s (relative to the sequence onset).**

**Fig S2. Correlation between PSE and probit-transformed (normalized) ITPCs at harmonic frequencies (4 and 6 Hz) across subjects.** No significant correlation was found in any of the conditions.

**Fig S3. Correlation between JND and probit-transformed (normalized) 2-Hz ITPCs across subjects.** No significant correlation was found in any of the conditions.

# Assessing the relation between PSE and ITPC using individual peak electrodes

The regression analysis reported in the main text used ITPC values extracted from a broader visual region which was the same across subjects (twenty-six electrodes; see Method). To address the possibility that the selection of such a broad ROI in the ITPC analysis might have missed individual differences across subjects in how ITPC values relate to PSE, we ran a supplementary analysis using individual peak electrodes per condition. In each flickering condition and for each subject, we identified the five electrodes showing the highest average ITPC peak during the time window of interest, that is, the entrainment period from 1 to 2 s after stimulus onset. Thus, the condition-specific individual peak electrodes were identified for 2 Hz (as well as independently for the 4 and 6 Hz harmonics for sake of exploratory analysis) and were averaged. Subsequently, identical to the analysis reported in the main text, we ran the additive mixed-effects regression model (PSE ~ condition + ITPC + (1|subject)) to test whether 2Hz-ITPC was related to PSE. Next, we compared the additive model with the condition × ITPC interaction model to examine whether the ITPC–PSE relationship differed across flickering conditions. Results of the additive regression model indicated that the main effect of ITPC was not significant (*b* = -0.076, SE = 0.107, 95% CI [−0.045, 0.535], *t*(37.99) = -0.70, *p* = .483) suggesting that 2-Hz ITPC did not significantly predict PSE after accounting for condition-related differences in PSE. Adding the condition × ITPC interaction did not significantly improve model fit compared with the additive model, *χ*²(2) = 4.88, *p* = 0.08. Therefore, the ITPC-PSE relationship was not differed across flickering conditions. Furthermore, the correlations between PSEs and ITPC values at 2 Hz and its harmonics (4 and 6 Hz) were not significant in any of the flickering conditions (Fig S4). These results suggest that even after accounting for individual peak electrode variations within subjects and conditions, flicker-induced time dilation remains unrelated to the magnitude of entrainment marked by ITPC.

**Fig S4. Correlation between PSE and probit-transformed (normalized) ITPC values across subjects, using individual peak electrodes for each condition**. ITPC values were extracted from the top five electrodes showing the highest ITPC for each subject and condition, independently for each frequency. No significant correlation was found in any condition.

# Category specific analysis

The primary motivation of this study was to assess how general entrainment of higher-level ventral regions, irrespective of category, affects time dilation compared with lower-level visual regions. Consequently, in the task design of this study, the same image was used to create the standard and comparison stimuli on each trial. This allowed both low-level visual and high-level semantic confounds to be controlled and canceled out in the relative judgment of duration. Thus, we did not expect to find any category differences based on the design of this study. Nevertheless, in an exploratory analysis, we sought to investigate if face versus house images have led to cataegory-specifc modulation of time perception, ITPC, and how time perception and ITPC relate.

## Behavioral results

For PSE, A 2 (category)×5 (condition) repeated-measures ANOVA showed no significant main effect of category, F(1, 18) = 0.14, p = .715, ηp² = .008, confriming that houses and faces did not affect PSEs differently. As expected, there was a significant main effect of condition, F(2.52, 45.29) = 20.96, p < .001, ηp² = .538. Moreover, the category × condition interaction was not significant, F(4, 72) = 0.60, p = .665, ηp² = .032.

Nevetheless, we followed up the condition effect seperately for each category to assess whether a same pattern of time dilation can be observed within each category (Fig S5). For faces, the post hoc t-test with bonferroni correction revealed that all conditions relative to the static condition resulted in significant time dilation effect (for scramble, t(18) = -5.03, *p* < 0.001, 95% CI [-0.623, -0.139], Cohen’s *d* = -1.35; for luminance flicker, t(18) = -5.20, *p* < 0.001, 95% CI [-0.722, -0.172], Cohen’s *d* = -1.58; for semantic flicker, t(18) = -4.44, *p* = 0.003, 95% CI [-0.608, -0.099], Cohen’s *d* = -1.25; for combined flicker, t(18) = -7.04, *p* < 0.001, 95% CI [-0.632, -0.237], Cohen’s *d* = -1.53). All other comparisons were insignificant (*p* = 1).

For houses, the post hoc tests revealed the same time dilation effect for all conditions relative to the static condition (for scramble, t(18) = -5.03, *p* < 0.001, 95% CI [-0.553, -0.123], Cohen’s *d* = -1.17; for luminance flicker, t(18) = -4.61, *p* = 0.002, 95% CI [-0.661, -0.120], Cohen’s *d* = -1.35; for semantic flicker, t(18) = -4.73, *p* = 0.002, 95% CI [-0.681, -0.131], Cohen’s *d* = -1.41; for combined flicker, t(18) = -5.33, *p* < 0.001, 95% CI [-0.638, -0.159], Cohen’s *d* = -1.38). All other comparisons were insignificant (*p* = 1).

For JND, A 2 (category)×5 (condition) repeated-measures ANOVA revealed no significant main effect of category, F(1, 18) = 0.03, p = .859, ηp² = .002, confirming the stumulus category did not change the temporal sensitivry across conditions (Fig S5). The main effect of condition was not significant, F(2.63, 47.25) = 2.24, p = .104, ηp² = .111. The category × condition interaction was also not significant, F(4, 72) = 0.56, p = .692, ηp² = .030.

Overall, the behavioral results indicated that stimulus category did not affect time perception. Moreover, the pattern of time dilation relative to the static condition was observed within each category.

**Fig S5. PSE and JND by category.** No main effect of category was found for both PSE and JND. Moreover, similar to the findings obtained with the full image set (reported in the main text), all conditions resulted in significant time dilation (PSE shift) relative to the static condition in both the face and house stimulus categories. JND was not significantly different in any of the conditions for both categories. Error bar indicates 95% confidence interval. * p<0.05, *** p<0.001.

## ITPC results

To analyze category-specific differences in ITPC, we focused only on the flickering conditions, in which the signal-to-noise ratio of the 2-Hz SSVEP was highest (compared to random noise in static and scramble conditions). The topographic maps showed that faces elicited stronger right temporoparietal entrainment than houses (Fig S6a). This is consistent with lateralization of face processing and previous SSVEP and SWIFT studies [1–5]. We analyzed the 2-Hz ITPC magnitide averaged over the pre-defined ROI (marked by dimands in Fig S6a) using A 2 (category)×3 (condition) repeated-measures ANOVA (Fig S6b). Results revealed no significant main effect of category, *F*(1, 18) = 1.93, *p* = .182, ηp² = .097. As expected, there was a significant main effect of condition, *F*(2, 36) = 9.86, *p* < .001, ηp² = .354. The category × condition interaction was not significant, *F*(2, 36) = 0.33, *p* = .724, ηp² = .018. Nevetheless, we followed up the condition effect seperately for each category.

For faces, the post hoc t-test with bonferroni correction revealed that combined flickers resulted in significantly higher ITPC relaive to luminance (t(18) = -2.88, *p* = 0.03, 95% CI [-0.247, -0.01], Cohen’s *d* = -0.87) and semantic flickers (t(18) = -3.10, *p* = 0.018, 95% CI [-0.272, -0.222], Cohen’s *d* = -0.10). Yet, the semantic and luminance flickers resulted in statisitcally same ITPC magnitude (t(18) = 0.44, *p* = 1, 95% CI [-0.09, 0.127], Cohen’s *d* = 0.12). For houses, post hoc tests revealed that combined flickers resulted in significantly higher ITPC relaive to luminance (t(18) = -2.86, *p* = 0.031, 95% CI [-0.255, -0.010], Cohen’s *d* = -0.9). Nevetheless, combined flickers higher ITPC magnitude relative to semantic flickers did not reach significance (t(18) = -2.33, *p* = 0.095, 95% CI [-0.227, 0.014], Cohen’s *d* = -0.72). Moreover, the semantic and luminance flickers resulted in statisitcally same ITPC magnitude (t(18) = -0.57, *p* = 1, 95% CI [-0.145, 0.093], Cohen’s *d* = -0.17).

Overall, the 2-Hz ITPC results obtained with the full image set (Fig 4b) were also replicated within each image category (Fig S6b). Moreover, we did not find any singifincat correlation between 2-Hz ITPC and PSE or JND for face (Fig S7) and house stimuli (Fig S8).

**Fig S6. 2-Hz ITPC by category.** (a) topographic maps of 2-Hz ITPC for face stimuli, house stimuli, and the face-versus-house contrast. Face stimuli resulted in lateralized left temporoparietal entrainment compared to house stimuli. Diamond markers indicate the pre-defined ROI same as main text. (b) the ITPC magnitude averaged over the ROI was not significantly different across face and house categories. Error bar indicates 95% confidence interval.

**Fig S7. Correlations between PSE and probit-transformed 2-Hz ITPC values, and between JND and probit-transformed 2-Hz ITPC values, across subjects for face stimuli.** No significant correlation was found in any of the conditions.

**Fig S8. Correlations between PSE and probit-transformed 2-Hz ITPC values, and between JND and probit-transformed 2-Hz ITPC values, across subjects for house stimuli.** No significant correlation was found in any of the conditions.

# Addressing the possibility of a saturation effect

To formally investigate if the scrambling present in the experimental conditions has lead to the saturation of time dilation effect, we conducted a supplmentary analysis. First, for each subject, we calculated the standard deviaton of PSEs across the four experimental conditons of scramble, luminance, semantic, and combined flickers. The ditribution of within-subject standard deviation of PSEs across the aforementioned conditions is shown in Fig S3. Based on this distribution, we set a moderate threshold of 0.1 seconds and subjects with a standard deviation below this threshold were excluded from the analysis. Thus, among the remaining 12 out of 19 participants, 68% of the PSEs across the specified conditions fell at least within ±0.1-seconds range around participant's mean. This ensured that any potential saturation was effectively controlled for the retained subjects who had greater within-subject variance across scramble and flickering conditions. We applied rANOVA to the PSEs of this selected subset of subjects. The post hoc comparison of PSEs across conditions for this subset of subjects is provided in Table S1. For comparsion, the post hoc comparison of PSEs for the whole sample is provided in Table S2. As it is evident, the pattern of significance did not change for the selected subset relative to the whole sample. Importantly, the Bayes factors of null difference across experimental conditions were comparable to those obtained from the whole sample.

Moreover, we assessed the correlation between PSEs and 2-Hz ITPC values across this selected subset of subjects. Nevertheless, no significant correlation was found (Fig S4).

**Fig S9. Distribution of within-subject standard deviation of PSE (n = 19) across the four experimental conditions of scramble, luminance, semantic, and combined flickers.** Red dashed line indicates the cut-off threshold (0.1 seconds) used to retain subjects whose PSEs across experimental conditions were less susceptible to saturation (see text).

**S1 Table. The post hoc comparison of PSE acorss conditions for the saturation-free subset of subjects (n = 12).** The highlighted Bonferroni-corrected p-values indicate the significant PSE differences across conditons. The highlighted Bayes factor values indicate contrasts which had null effect.

| ***Post Hoc Comparisons*** | | | | | |  |
| --- | --- | --- | --- | --- | --- | --- |
|  |  | **Mean Difference** | **SE** | **t** | **p_bonf_** | **BF_01_** |
| **Static** | **Scramble** | -0.272 | 0.077 | -3.534 | **0.047** | 0.091 |
|  | **Luminance Flicker** | -0.337 | 0.099 | -3.412 | **0.058** | 0.108 |
|  | **Semantic Flicker** | -0.301 | 0.085 | -3.545 | **0.046** | 0.089 |
|  | **Combined Flicker** | -0.356 | 0.077 | -4.598 | **0.008** | 0.020 |
| **Scramble** | **Luminance Flicker** | -0.065 | 0.081 | -0.807 | 1.000 | **2.640** |
|  | **Semantic Flicker** | -0.029 | 0.064 | -0.454 | 1.000 | **3.183** |
|  | **Combined Flicker** | -0.084 | 0.067 | -1.264 | 1.000 | **1.814** |
| **Luminance Flicker** | **Semantic Flicker** | 0.036 | 0.050 | 0.731 | 1.000 | **2.771** |
|  | **Combined Flicker** | -0.019 | 0.056 | -0.339 | 1.000 | **3.310** |
| **Semantic Flicker** | **Combined Flicker** | -0.055 | 0.060 | -0.925 | 1.000 | **2.427** |
|  | | | | | |  |
| *Note.*  P-value adjusted for comparing a family of 10 | | | | | |  |

**S2 Table. The post hoc comparison of PSE across conditions obtained from the whole sample (n = 19) reported in the main text.** The highlighted Bonferroni-corrected p-values indicate the significant PSE differences. The highlighted Bayes factor values indicate contrasts which had null effect.

| ***Post Hoc Comparisons*** | | | | | |  |
| --- | --- | --- | --- | --- | --- | --- |
|  |  | **Mean Difference** | **SE** | **t** | **p_bonf_** | **BF_01_** |
| **Static** | **Scramble** | -0.358 | 0.063 | -5.660 | **< .001** | 9.505×10^-4^ |
|  | **Luminance Flicker** | -0.412 | 0.075 | -5.490 | **< .001** | 0.001 |
|  | **Semantic Flicker** | -0.379 | 0.070 | -5.448 | **< .001** | 0.001 |
|  | **Combined Flicker** | -0.411 | 0.060 | -6.786 | **< .001** | 1.216×10^-4^ |
| **Scramble** | **Luminance Flicker** | -0.054 | 0.052 | -1.050 | 1.000 | **2.597** |
|  | **Semantic Flicker** | -0.022 | 0.044 | -0.492 | 1.000 | **3.777** |
|  | **Combined Flicker** | -0.053 | 0.046 | -1.156 | 1.000 | **2.352** |
| **Luminance Flicker** | **Semantic Flicker** | 0.033 | 0.034 | 0.972 | 1.000 | **2.779** |
|  | **Combined Flicker** | 0.002 | 0.038 | 0.043 | 1.000 | **4.207** |
| **Semantic Flicker** | **Combined Flicker** | -0.031 | 0.039 | -0.800 | 1.000 | **3.171** |
|  | | | | | |  |
| *Note.*  P-value adjusted for comparing a family of 10 | | | | | |  |

**Fig S10. Correlation between PSE and normalized (probit-transformed) 2-Hz ITPC values across the saturation-free subjects (n = 12).** No significant correlation was found in any of the conditions.

# Event-related potential (ERP) analysis

In a supplementary analysis, we focued on assessing whether the amplitude of ERP components implied in time perpection studies is significantly different among conditions. We focued on contingent negative variation (CNV) and P2 ampltude based on a previous study [6]. For this purpose, the single-trial EEG data were averaged over Cz, and three other electrodes roughly corresponding to FCz, FC1 and FC2 on our 64 HydroCel GSN cap (the selected electrodes’ positions are shown in Fig S5). CNV was defined as the average amplitude over 0.1 s time window immediately before standard stimuli’s offset (2.4 – 2.5 s, dark gray shaded area in Fig S5) in each condition [7]. P2 was defined as the average amplitude within 2.7 – 2.85 s time window (light gray shaded area in Fig S5). Prior to signal averaging, in each condition and for each subject, P2 amplitude was baseline corrected to the average of amplitudes at 2.45 – 2.55 s time window [6].

rANOVA both on CNV (F (4,72) = 1.06, *p* = 0.37) and P2 (F (4,72) = 0.55, *p* = 0.7) was insignificant. Thus, in terms of ERP amplitudes, as shown in Fig S5, no significant difference among conditions was found. Moreover, the amplitude of the CNV and P2 did not show significant correlation with PSE in any of the conditions (data not shown)

**Fig S11. ERP analysis. The dark shaded area indicates the time window defined for analysis of CNV.** The light gray shaded area represents the time window chosen for analysis of P2 amplitude. The head plot shows the selected electrodes across which the ERP waves were averaged.

# Time frequency power analysis

To assess whether there were any power dynamic differences in induced neural oscillations across conditions, we conducted time frequency power analysis. For this purpose, similar to ITPC, we used complex Morlet wavelets at frequencies from 1 to 30 Hz in steps of 0.5 Hz using custom-written MATLAB scripts [8]. The number of wavelet cycles was set to the logarithmically spaced range of 4 to 10 to balance the time-frequency precision [8]. The significance of clusters was assessed using the exact permutation statistical procedure reported in the main text. Results are shown in Fig S6. In flickering conditions, a broadband desynchronization was observed in alpha and beta frequencies which was absent for the scrambling conditions. Nevertheless, no significant major cluster was detected in any of the conditions relative to the static baseline condition.

**Fig S12. Time frequency power analysis.** No significant cluster was detected relative to the static baseline condition.

# References

1.	Alonso-Prieto E, Belle G Van, Liu-Shuang J, Norcia AM, Rossion B. The 6 Hz fundamental stimulation frequency rate for individual face discrimination in the right occipito-temporal cortex. Neuropsychologia. 2013;51: 2863–75. doi:10.1016/j.neuropsychologia.2013.08.018

2.	Bentin S, Sagiv N, Mecklinger A, Friederici A, von Cramon YD. Priming visual face-processing mechanisms: electrophysiological evidence. Psychol Sci. 2002;13: 190–3. doi:10.1111/1467-9280.00435

3.	Rossion B. Understanding face perception by means of human electrophysiology. Trends Cogn Sci. 2014;18: 310–8. doi:10.1016/j.tics.2014.02.013

4.	Lueschow A, Sander T, Boehm SG, Nolte G, Trahms L, Curio G. Looking for faces: Attention modulates early occipitotemporal object processing. Psychophysiology. 2004;41: 350–60. doi:10.1111/j.1469-8986.2004.00159.x

5.	Gagsch F, Valuch C, Albrecht T. Measuring attentional selection of object categories using hierarchical frequency tagging. J Vis. 2024;24: 8. doi:10.1167/jov.24.7.8

6.	Kononowicz TW, van Rijn H. Decoupling interval timing and climbing neural activity: a dissociation between CNV and N1P2 amplitudes. J Neurosci. 2014;34: 2931–9. doi:10.1523/JNEUROSCI.2523-13.2014

7.	Boehm U, van Maanen L, Forstmann B, van Rijn H. Trial-by-trial fluctuations in CNV amplitude reflect anticipatory adjustment of response caution. Neuroimage. 2014;96: 95–105. doi:10.1016/j.neuroimage.2014.03.063

8.	Cohen MX. Analyzing Neural Time Series Data. Analyzing Neural Time Series Data. The MIT Press; 2014. doi:10.7551/mitpress/9609.001.0001
