# Supplementary material for: Toward understanding the role of visual hierarchy in flicker-induced time dilation: A preregistered study
Source: PLoS One. 2026 Oct 13;21(10):e0337307. doi: 10.1371/journal.pone.0337307 (PMC13660902; doi:10.1371/journal.pone.0337307)
Supplement: Supplementary file 2 [file pone.0337307.s003.docx]

We wish to express our appreciation to the editor and the reviewers for their insightful comments, which have helped us significantly improve our manuscript. We hope that the revisions improve the paper such that the editor and the reviewers now deem it worthy of publication in PLOS ONE.

Next, we offer detailed responses to the editor and reviewers’ comments. The editor and reviewers’ comments are shown in BOLD font, our responses are in blue, and the sentences in the revised manuscript and the supplementary file are shown in italic. All the changes to the manuscript are highlighted by the yellow color.

**Dear Dr. Houshmand Chatroudi,

Thank you for submitting your manuscript to PLOS ONE. After careful consideration, we feel that it has merit but does not fully meet PLOS ONE’s publication criteria as it currently stands. Therefore, we invite you to submit a revised version of the manuscript that addresses the points raised during the review process.

Two expert Reviewers evaluated the manuscript and they acknowledge it is well-done and well-described. Nevertheless, they also suggest some limitations and I would like to ask you to consider all the suggestions received, with a particular focus on ITPC analysis and EEG filters (as pointed out by Reviewer 1) and on the explanation of the low repetitions (yielding low power) and of the behavioral results, together with the adding of some more details of the results (e.g., effect sizes and confidence intervals for all results), as suggested by Reviewer 2.**

We would like to thank you and the reviewers for the diligent reading of our manuscript and for the constructive comments provided. We believe that our manuscript has greatly benefited from these suggestions, which have improved its clarity and readability. Thanks to Reviewer 1’s comment, in addition to our main analysis focused on a broader region of interest (ROI), we have conducted supplementary analysis using individual peak electrodes per condition. The results of this analysis were not different from what was originally reported in the manuscript which further strengthened the findings. Additionally, we have improved the description of wavelet (filter) decomposition method we have used for time-frequency analysis. Moreover, thanks to Reviewer 2’s comment, we have added a subsection titled ‘Limitations’ to the Discussion in which we explicitly highlight the limitations of this study. In short, the present study showed clear evidence of entrainment in the EEG data. However, we did not find evidence that time dilation was related to entrainment, either across the experimentally manipulated conditions or within-condition correlations. Therefore, in addition to the conjectures already offered in the Discussion, we have now discussed the constraints of the current study design. Nevertheless, we have provided cogent arguments and additional supplementary analyses explaining that the low number of repetitions in this study did not affect the main findings reported in the manuscript (see our reply to Reviewer 2). Lastly, we have improved the clarity of the manuscript by revising vague terms and adding confidence intervals and effect sizes. We hope that our modifications have satisfactorily addressed the concerns of the Editor and reviewers.

**Please include the following items when submitting your revised manuscript:**

- **A letter that responds to each point raised by the academic editor and reviewer(s). You should upload this letter as a separate file labeled 'Response to Reviewers'.**
- **A marked-up copy of your manuscript that highlights changes made to the original version. You should upload this as a separate file labeled 'Revised Manuscript with Track Changes'.**
- **An unmarked version of your revised paper without tracked changes. You should upload this as a separate file labeled 'Manuscript'.**

**If you would like to make changes to your financial disclosure, please include your updated statement in your cover letter. Guidelines for resubmitting your figure files are available below the reviewer comments at the end of this letter.

We look forward to receiving your revised manuscript.**

**Kind regards,**

**Giulia Prete
Academic Editor
PLOS One**

**Journal Requirements:**

**When submitting your revision, we need you to address these additional requirements.**

**1. Please ensure that your manuscript meets PLOS ONE's style requirements, including those for file naming. The PLOS ONE style templates can be found at**

Thank you verry much. We have ensured that our manuscript meets PLOS ONE style requirements.

**2. We note that you have included the phrase “data not shown” in your manuscript. Unfortunately, this does not meet our data sharing requirements. PLOS does not permit references to inaccessible data. We require that authors provide all relevant data within the paper, Supporting Information files, or in an acceptable, public repository. Please add a citation to support this phrase or upload the data that corresponds with these findings to a stable repository (such as Figshare or Dryad) and provide and URLs, DOIs, or accession numbers that may be used to access these data. Or, if the data are not a core part of the research being presented in your study, we ask that you remove the phrase that refers to these data.**

Thank you very much. We have added the required figure (Figure S2 in S1 Appendix) and have properly cited it in the manuscript.

**3. We note that Figures 1, 2 and Movie S1 in Supporting information file includes an image of a participant  in the study.**

**As per the PLOS ONE policy (**[**http://journals.plos.org/plosone/s/submission-guidelines#loc-human-subjects-research**](http://journals.plos.org/plosone/s/submission-guidelines#loc-human-subjects-research)**) on papers that include identifying, or potentially identifying, information, the individual(s) or parent(s)/guardian(s) must be informed of the terms of the PLOS open-access (CC-BY) license and provide specific permission for publication of these details under the terms of this license. Please download the Consent Form for Publication in a PLOS Journal (**[**http://journals.plos.org/plosone/s/file?id=8ce6/plos-consent-form-english.pdf**](http://journals.plos.org/plosone/s/file?id=8ce6/plos-consent-form-english.pdf)**). The signed consent form should not be submitted with the manuscript, but should be securely filed in the individual's case notes. Please amend the methods section and ethics statement of the manuscript to explicitly state that the patient/participant has provided consent for publication: “The individual in this manuscript has given written informed consent (as outlined in PLOS consent form) to publish these case details”.**

**If you are unable to obtain consent from the subject of the photograph, you will need to remove the figure and any other textual identifying information or case descriptions for this individual.**

Thank you for bringing this issue to our attention. As it specified in the method section of the manuscript, sixteen free-to-use images were found on Pexels website (https://www.pexels.com/) and were subsequently used in this research (including the exemplary female photograph that is used partly in Fig 1 & Fig 2). Our approach is similar to previous published papers (e.g., https://journals.plos.org/plosone/article?id=10.1371/journal.pone.0144858). Regarding the exemplary female photograph used partly in Fig 1 & Fig 2, we humbly ask you to refer to the source image website (https://www.pexels.com/photo/woman-wearing-blue-top-2169434/). As it is noted on the left bottom side of the image, this photo is a Free to use image. The license policies of pexels website (https://www.pexels.com/license/) clearly state that these images are free to be used/edited without attribution.

Nevertheless, to comply with your strict requirements, we have replaced the image with the image of the first author of this manuscript (who has signed the PLOS ONE consent form to publish his image in this paper) and have properly mentioned in the ethic statement section that this image is used only for displaying purposes and has not been among the stimuli set used in the experiment.

On lines 162-167:

*“Moreover, the image of the individual shown in Figures 1 and 2, as well as in the supplementary movies (S1 Appendix), is included only for display purposes and does not correspond to any of the actual face images used in the experiment because of copyright and publication restrictions. The individual in this manuscript has given written informed consent (as outlined in PLOS consent form) to publish these case details.”*

**4. Please include captions for your Supporting Information files at the end of your manuscript, and update any in-text citations to match accordingly. Please see our Supporting Information guidelines for more information:** [**http://journals.plos.org/plosone/s/supporting-information**](http://journals.plos.org/plosone/s/supporting-information)**.**

Thank you for pointing out this issue. Since our supplementary materials include multiple analyses and results, we have integrated all of them into one file named ‘S1 Appendix’. Therefore, all supplementary images in this unified file are referred to accordingly. For example, when referring to the second supplementary figure, we have phrased it as ‘Fig S2 in S1 Appendix’, or when referring to supplementary movies, ‘Movie S1 in S1 Appendix’.

**5. If the reviewer comments include a recommendation to cite specific previously published works, please review and evaluate these publications to determine whether they are relevant and should be cited. There is no requirement to cite these works unless the editor has indicated otherwise.**

Thank you very much. We have acted accordingly.  **Reviewers' comments:**

**5. Review Comments to the Author**

**Reviewer #1: This pre-registered study tests whether subjective time dilation induced by visual flicker results from neural entrainment in low or high level visual area. The design cleverly combines basic luminance and semantic flicker (same low-level image properties, but periodic occurrence of a face or house) in a forced choice interval duration comparison task.
The EEG data show robust phase clustering by the flickering stimuli both for luminance and semantic flickers, hence clear evidence for phase entrainment.
The behavioral data show no specific time dilation with respect to the flicker, as all conditions, including the scrambled one are dilated compared to the static one. A small difference is observed in JND, but neither the PSD nor the JND effects correlate with the phase entrainment quantified by the ITC.
The study design and analyses are very clear. Despite the null effect between entrainment and duration dilation, this work is a valuable addition to the literature; its methodological rigor provides strong evidence for the lack of a relationship. I also found the discussion particularly compelling, offering insightful arguments to contextualize the results.**

We would like to express our sincere appreciation to Reviewer 1 for his constructive suggestions, which improved the clarity of the manuscript and the analysis. Specifically, based on your suggestions, we ran a supplementary control analysis using individual peak electrodes in each flickering condition. The null results remained unchanged, strengthening the findings obtained from the main analysis reported in the manuscript. This analysis and its results are now included in the supplementary materials (S1 Appendix). Moreover, thanks to your comment, we reformulated the analysis addressing the relationship between PSE and ITPC as a clear model-based regression analysis. Lastly, we implemented all the clarifications you requested in the Methods section, which significantly improved the readability of the manuscript.

**Regarding the methodology, the ITPC analysis was quite broad (using the same electrodes for all participants, averaged over a fixed window). Would extracting ITPC from individual peak electrodes, and perhaps even per condition, allow for a more refined assessment of entrainment and its potential links to timing behavior?**

Thank you very much for suggesting this control analysis. In addition to the analysis reported in the main text which was focused on an identical broader visual region across subjects (twenty-six electrodes, see Method), we ran a supplementary analysis focusing on individual peak electrodes per condition.

Based on your suggestion, in each flickering condition and per subject, we identified the top five electrodes that showed the highest average ITPC peak during the time window of interest (i.e., the entrainment period which is 1 to 2-second time window after stimulus onset). The condition specific individual peak electrodes were independently identified for 2 Hz as the main frequency of manipulation and for the first (4 Hz) and second harmonics (6 Hz) for the sake of exploration.

Subsequently, identical to the analysis reported in the main text, we ran the additive mixed-effects regression model (PSE ~ condition + ITPC + (1|subject)) to test whether 2Hz-ITPC was related to PSE. Next, we compared the additive model with the condition × ITPC interaction model to examine whether the ITPC–PSE relationship differed across flickering conditions.

Results of the additive regression model indicated that the main effect of ITPC was not significant (*b* = -0.076, SE = 0.107, 95% CI [−0.045, 0.535], *t*(37.99) = -0.70, *p* = .483) suggesting that 2-Hz ITPC did not significantly predict PSE after accounting for condition-related differences in PSE. Adding the condition × ITPC interaction did not significantly improve model fit compared with the additive model, χ²(2) = 4.88, p = 0.08. Therefore, the ITPC-PSE relationship was not differed across flickering conditions. Furthermore, the correlations between PSEs and ITPC values at 2 Hz and its harmonics (4 and 6 Hz) were not significant in any of the conditions (Fig S4). This result suggests that even after accounting for individual peak electrode variations within subjects and conditions, flicker time dilation stays unrelated with the magnitude of entrainment marked by ITPC. This control analysis is now added to the supplementary information and is briefly mentioned in the main text.

In the main text on lines 584-588,

*“This lack of correlation was also observed between PSE and ITPC values at the harmonic frequencies (Fig S2 in S1 Appendix) and remained unchanged even after restricting the analysis to ITPC values derived from individual peak electrodes within conditions (Fig S4 in S1 Appendix).”*

In the supplementary information a new section is created:

“*Assessing the relation between PSE and ITPC using individual peak electrodes*

*The regression analysis reported in the main text used ITPC values extracted from a broader visual region which was the same across subjects (twenty-six electrodes; see Method). To address the possibility that the selection of such a broad ROI in the ITPC analysis might have missed individual differences across subjects in how ITPC values relate to PSE, we ran a supplementary analysis using individual peak electrodes per condition. In each flickering condition and for each subject, we identified the five electrodes showing the highest average ITPC peak during the time window of interest, that is, the entrainment period from 1 to 2 s after stimulus onset. Thus, the condition-specific individual peak electrodes were identified for 2 Hz (as well as independently for the 4 and 6 Hz harmonics for sake of exploratory analysis) and were averaged. Subsequently, identical to the analysis reported in the main text, we ran the additive mixed-effects regression model (PSE ~ condition + ITPC + (1|subject)) to test whether 2Hz-ITPC was related to PSE. Next, we compared the additive model with the condition × ITPC interaction model to examine whether the ITPC–PSE relationship differed across flickering conditions. Results of the additive regression model indicated that the main effect of ITPC was not significant (b = -0.076, SE = 0.107, 95% CI [−0.045, 0.535], t(37.99) = -0.70, p = .483) suggesting that 2-Hz ITPC did not significantly predict PSE after accounting for condition-related differences in PSE. Adding the condition × ITPC interaction did not significantly improve model fit compared with the additive model, χ²(2) = 4.88, p = 0.08. Therefore, the ITPC-PSE relationship was not differed across flickering conditions. Furthermore, the correlations between PSEs and ITPC values at 2 Hz and its harmonics (4 and 6 Hz) were not significant in any of the flickering conditions (Fig S4). These results suggest that even after accounting for individual peak electrode variations within subjects and conditions, flicker-induced time dilation remains unrelated to the magnitude of entrainment marked by ITPC.*

***Fig. S4. Correlation between PSE and probit-transformed (normalized) ITPC values across subjects, using individual peak electrodes for each condition****. ITPC values were extracted from the top five electrodes showing the highest ITPC for each subject and condition, independently for each frequency. No significant correlation was found in any condition.”*

**To clarify:

Where the flicker conditions all intermixed on a trial by trial basis?**

We appreciate for helping us notice that this point was vague in the manuscript. We fixed the wording to make it clear that the conditions were intermixed on a trial-by-trial basis.

On lines 315 – 318:

“*A total of 560 randomized trials were divided into seven blocks, with all conditions intermixed on a trial-by-trial basis. Six mandatory rest periods were provided between blocks, each lasting 5 to 8 minutes. The experiment lasted approximately three hours.*.”  **Report details of EEG filters, which are especially relevant for the phase analysis.**

Thank you for bringing this point to our attention. The wavelet decomposition method used in our analysis closely followed the approach described in Analyzing Neural Time Series Data: Theory and Practice [1]. We have added the following details to clarify the properties of the wavelet filters used in the phase analysis.

On lines 367-373:

“*The Gaussian width was defined as s=n/(2πf), where n is the number of cycles and f is the center frequency. The temporal full width at half maximum (FWHM) of the wavelets ranged from approximately 2.25 s at 1 Hz to 0.30 s at 10 Hz, and the spectral FWHM ranged from approximately 0.39 Hz at 1 Hz to 2.94 Hz at 10 Hz. Convolution was performed in the frequency domain using FFT. The resulting complex signal was used to extract phase values (for detailed description of this method, see [68]).*  ”

**EEG epochs: with respect to which events were the epochs created? Only for the comparison stimuli?**

Thank you for your comment. We realized that this point was not sufficiently clear; therefore, we rephrased this section to improve clarity. In this study, the experimental conditions were assigned to the standard stimuli (Fig 2), which ensured an equal duration of 2.5 s across all conditions. Consequently, epochs were created using only the standard stimuli.

On lines 350-353:

*“In this study, the experimental conditions were assigned to the standard stimuli (Fig 2), which ensured equal duration across all conditions. Consequently, epochs were created using only the standard stimuli.”*

**Was an extra baseline correction applied to the time-frequency transforms or ITPC values?**

We appreciate your comment. No additional baseline correction was applied to the time frequency transforms or the ITPC values. Unlike power-based time frequency transforms, ITPC values do not always need baseline correction [1]. To clarify the procedure, we added the following sentence on line 380:

*“….. discarding the ITPC values at the beginning and end of the epochs [42] (No baseline correction was applied).*”

**p.17, l. 379-380: what is meant by 'time window region of interest'?**

We sincerely apologize for our poor choice of wording. We have revised these lines to improve clarity.

On lines 385-391:

***“…*** *we limited the ITPC analysis to a broad visual region of interest (ROI) comprising occipital, parietal, and temporal regions. Specifically, ITPC values were averaged across the following channels: E16, E20, E21, E22, E25, E26, E27, E28, E31, E33, E34, E35, E36, E37, E38, E39, E40, E41, E42, E45, E46, E48, E49, E50, E51, and Cz (Fig 4b). For each subject, the ITPC values at 2 Hz were averaged across the selected channels and over the 1- to 2-second time window relative to stimulus onset.****”***

**Explain why the probit transform of ITPC values was performed in the main manuscript so the reader does not have to go back and forth between the preregistration and the paper.**

We appreciate you for pointing this out. We have rephrased the section to briefly mention why probit-transform was necessary.

On lines 391-395:

***“****Next, these average values were probit-transformed. Because ITPC values are bounded between 0 and 1, they were probit-transformed using the inverse cumulative distribution function to better approximate normality [68]. The transformed values were then subjected to a one-way repeated-measures analysis of variance (rANOVA).****”*

p. 20, l.414 – 422: This could be formulated more clearly. Does the analysis check for main effects of condition and ITPC on behavior (DV)?**

We are truly thankful for helping us notice this negligence. We have reformulated the analysis and consequently have rephrased both the description and report of results to improve clarity.

The description of this analysis is now reformulated in a clearer way on lines 426-436:

***“****Moreover, to examine whether ITPC was generally associated with PSE across conditions, we first fitted an additive mixed-effects linear regression model with condition and probit-transformed ITPC as fixed effects and subjects as random effects. In this model, the main effect of ITPC tested whether ITPC predicted PSE after accounting for condition-related differences in PSE. We then compared the additive model against the condition × ITPC interaction regression model to examine whether the ITPC–PSE relationship differed across conditions. If a significant interaction was found, post hoc single regressions between ITPC and PSE were performed separately for each condition, with false discovery rate* [2] *applied for multiple comparison corrections. The same analysis was conducted for JND to assess ITPC and JND relationship. ”*

The result of this reformulated analysis is reported on lines 577-583 for PSE:

*“Moreover, results of the additive regression model indicated that the main effect of ITPC was not significant (b = 0.245, SE = 0.148, 95% CI [−0.045, 0.535], t(78.64) = 1.66, p = .101) suggesting that 2-Hz ITPC did not significantly predict PSE after accounting for condition-related differences in PSE. Adding the condition × ITPC interaction did not significantly improve model fit compared with the additive model (χ²(4) = 4.75, p = .314). Therefore, there was no evidence that the relationship between 2-Hz ITPC and PSE differed across conditions.”*

And on lines 600-606 for JND:

*“Lastly, the additive regression model with JND as the dependent variable showed that the main effect of ITPC was not significant (b = -0.004, SE = 0.075, 95% CI [−0.152, 0.144], t(76.37) = -0.05, p = .956). This suggests that 2-Hz ITPC did not significantly predict JND after accounting for condition-related differences in PSE. Adding the condition × ITPC interaction did not significantly improve model fit compared with the additive model (χ²(4) = 5.08, p = .279). Therefore, these results point to an absence of a relationship between temporal sensitivity and entertainment (Fig S3 in S1 Appendix).”*
 **Minor:

p.6, l.112: 'The ventral stream of the vision' -> in vision
p. 9, l.182 using histogram equalization function -> 'a histogram'..
p. 25, l. 566 Bayse factor -> Bayes factor (also occurs at least once in the Discussion)**

We sincerely apologize for overlooking these grammatical and spelling errors in the manuscript. In addition to correcting the issues highlighted by you, we re-checked the manuscript to ensure that no further grammatical or spelling errors remain.

**Figure resolution appears low, but might be due to automatic pdf conversion?**

Thank you very much for bringing this issue to our attention. While the pictures we provided for this manuscript have passed the automatic quality check recommended by PLOS ONE (i.e., NAAS), we are unsure as to why they appear to have low-resolution in the final pdf. We will correspond with the Editor regarding this issue.

**Reviewer #2: This manuscript reports a preregistered experiment examining the cortical underpinnings of flicker-induced time dilation (FITD), specifically whether higher-level ventral visual stream regions contribute via selective neural entrainment using semantic wavelet-induced frequency tagging (SWIFT). The study integrates sophisticated stimulus generation with a robust psychophysical duration comparison task, yielding clear evidence of time dilation across dynamic conditions relative to static baselines, while finding no predicted modulation by flicker hierarchy. Although the preregistration and open science practices are commendable, the manuscript would benefit from a more cautious interpretation of the null and near-null findings. The work aligns well with PLOS ONE's emphasis on rigorous, transparent methodology in perception research.**

We would like to express our sincere appreciation to you for the meticulous reading of our paper and insightful and constructive suggestions that you have made. We believe that your comments have substantially benefited our manuscript by improving its clarity and helping us more clearly define its interpretational limitations. However, we have provided cogent arguments and supplementary data analyses demonstrating that the low number of repetitions of each image per condition-duration combination did not affect the main findings of this manuscript. Nevertheless, thanks to your comment, we have added a new section to the Discussion to explicitly address the limitations of this study. Moreover, your suggestions led us to perform supplementary category-specific analysis which further demonstrated that the findings observed with the full stimuli set were also present within each category. Lastly, thanks to your painstaking reading of our manuscript, we have clarified all vague expressions and have added confidence intervals and effect sizes. We hope that our modifications have adequately addressed your concerns.

**1. Strengths in Preregistration and Open Science Practices
The authors adhered closely to their detailed preregistration, which specifies hypotheses, experimental conditions (static, scramble, luminance flicker, semantic flicker, combined flicker at 2 Hz tagging frequency), sample size (N=21 targeted, N=20 after preregistered exclusion for poor temporal sensitivity), analysis pipeline (Palamedes psychometric fits with 2% lapse rates, repeated-measures ANOVA on PSE/JND, Bayes factors via JASP, ITPC correlations), and exclusion criteria. This fidelity reduces concerns about flexibility in data analysis and enhances credibility. Exploratory analyses (e.g., harmonics, power spectra, ERPs) are transparently flagged as such, avoiding HARKing. Raw data, scripts, and supplementary materials are publicly available on OSF, including stimulus movies—exemplary for reproducibility. The power analysis (targeting d=0.8 for ANOVA) is justified and reported, with post-hoc sensitivity noted.

2. Behavioral Methods and Task Design
The core behavioral paradigm—a 2-interval forced-choice (2IFC) duration discrimination task—is methodologically strong. Standard stimuli (2.5s fixed duration) in each of the five conditions are precisely matched to their static comparison stimuli (1-4s in 0.5s steps, same source image), effectively controlling for low-level (luminance, contrast, pixel modulation spectra) and high-level (semantic content: faces/houses) confounds between intervals. This within-trial matching is a key strength, isolating flicker manipulations from adaptation or image-specific biases. SWIFT normalization (CLAHE contrast, pixel FFT flattening to 7.2 Hz cutoff, frame-wise luminance equating) rigorously equates non-target features across semantic/scramble conditions, while in-phase luminance overlay in combined flickers tests hierarchical tagging as intended.
Trial structure minimizes confounds since randomized fixation/blank/ISI durations prevent counting. Moreover, instructions emphasize no counting and eye fixation. Psychometric fits yield PSE (bias) and JND (precision) as intuitive metrics for FITD magnitude and temporal sensitivity cost.**

Thank you very much for your meticulous reading of our manuscript, your positive evaluation and your support for open science practices.

**As for blocks structure, on one hand single repetition per image-duration-condition (560 trials over 7 blocks with rests) prevents excessive participants' fatigue. On the other hand, a potential flaw may arise in such low repetitions (yielding low power): with 16 images and only 1 repetition per image-duration-condition combination, image-specific noise is uncontrolled.**

Thank you very much for raising this important issue. We recognize the concern that the images were presented only once per condition-duration combination because of the lengthy duration of the experiment (three hours), and we agree that, from a methodological perspective, the ideal design would include several repetitions of each image per condition-duration combination. Therefore, we have reflected this concern in the limitation section of this manuscript.

On lines 826-830:

*“Moreover, because of the lengthy duration of the experiment, we presented each image only once per condition-duration combination. Although this limitation did not affect the EEG results, from a methodological perspective, future studies should include multiple repetitions of each image per condition-duration combination.”*

However, we do not believe that image-specific noise resulted in a loss of power for the main conclusion of this study or affected the estimation of time perception metrics (PSE and JND) or EEG entrainment.

In this study, we obtained clear EEG results showing all flickering conditions (compared to the static and scramble condition) resulted in robust phase clustering at the fundamental frequency and the harmonics (Fig 4 & 5). Importantly, the semantic condition resulted in the lateralized activation of right temporoparietal regions that is consistent with previous studies using faces and houses [3–7]. Moreover, the combined flicker condition, which was expected to simultaneously activate lower- and higher-level visual regions, resulted in phase clustering that was larger in magnitude and broader in topography (Fig 4a). Had single-image noise confounded our results, we would have failed to obtain a clear entrainment profile. However, as is evident from our findings, this was not the case. Nevertheless, we found that time dilation was not related to entrainment in this study. These results are discussed in detail, and in addition to the conjectures already offered in the Discussion, we have now highlighted the constraints of the current study design.

Furthermore, In the duration comparison paradigm in time perception studies, on each trial the duration of the standard stimulus is compared against the comparison stimulus. In adapting this paradigm to our study, we used the same image to create the standard and comparison stimuli on each trial. Therefore, the portion of the noise that is related to the static low-level image properties and high-level semantic confounds are canceled in such relative judgements for each image per trial (El Greco logic [8,9]). Moreover, in the stimuli pre-processing step, we equalized all the images in terms of size, mean luminance, and contrast distributions. Thus, a proportion of low-level visual properties was controlled across all images before they were input into the SWIFT algorithm, making image-specific noise comparable across stimuli. Furthermore, SWIFT algorithm was applied equally to all images (three harmonics were used and the level of decomposition was nine). Therefore, the aggregated individual pixel-frequency spectra were equalized in amplitude and cut off at 7.2 Hz for all images (Fig 3). Thus, the modulation of individual pixels across frames created a white-noise-like pattern that was comparable across all images. Therefore, attempts were made to strictly equalize all images in terms of both static and dynamic noise, leaving little space for single image noises to confound the findings resulting in loss of power.

However, we agree that some image properties, such as higher-order statistics, differed across images. Nevertheless, the assumption that one repetition per condition-duration combination resulted in a loss of power requires that image-specific noise interacted with both condition and duration. To test this possibility, we took advantage of your suggestion and conducted a category-based analysis of the time perception metrics. The face and house images differ systematically in higher-order image properties, such as spatial-frequency content, Fourier amplitude spectra, and orientation energy, with houses often containing stronger cardinal orientations and higher spatial-frequency edge structure [10–13]. Thus, if image-specific noise interacted with duration or condition, one would expect the time perception metrics to differ between these two categories, assuming that these categories served as a controlled source of SWIFT-related dynamic noise. Nevertheless, we found no main effect of category (for PSE, F(1, 18) = 0.14, *p* = .715, ηp² = .008; for JND, F(1, 18) = 0.03, p = .859, ηp² = .002) and neither a significant category × condition interaction [for PSE, F(4, 72) = 0.60, p = .665, ηp² = .032; for JND, F(4, 72) = 0.56, p = .692, ηp² = .030). Thus, the pattern of results observed with the combined stimulus set reported in the manuscript remained the same within each category (this analysis and its results are now included in the supplementary materials, see our reply to your next comment). Based on these results, we believe it is highly unlikely that image-specific noise reduced statistical power because of the lower number of repetitions per condition-duration combination.

Overall, the careful equalization of images in terms of both static and dynamic properties, together with the use of relative judgments on each trial, makes it unlikely that single-image noise acted as a confounding factor. Yet, to test if higher-order properties of images have confounded the results, we ran a category specific analysis. This was done because face and house images each share many of the higher-order image statistics within their categories. Thus, if noise derived from these properties had to reduce power, one would expect to see category differences in time perception metrics. However, no category influence was found, further confirming that much of single image noises were either controlled or canceled in the relative duration comparisons (also, we found no category differences in ITPC magnitude, see our reply to your next comment). Therefore, we believe it is highly unlikely that our design suffers from lack of power due to low repetition of each single image per condition-duration combination. Nevertheless, we agree with you that we should adopt a more cautious view in interpreting our findings and discuss the study design limitations. This is what we have done thanks to your comment (see our reply to **3. Key Behavioral Results and Interpretation**).

**Moreover, faces versus houses were selected based on fMRI motivation for ventral activation, yet no category analysis is reported despite the ventral stream hypothesis.**

The primary reason we selected faces and houses as stimuli was to assess how general entrainment of higher-level ventral regions, compared with lower-level visual regions, affects time dilation. Thus, we did not seek to conduct a category-specific analysis distinguishing how selective activation of ventral regions by faces versus houses affects time dilation. Therefore, the stimulus set was created to generalize the findings regarding the visual hierarchy irrespective of stimulus category (we specifically did not include objects due to their poor ability in activating higher-order ventral regions when used as SWIFT sequences [14]). Moreover, in our task design, the same image was used to create the standard and comparison stimuli. Hence, any effect of semantic content on time perception would be canceled out in the relative judgment of duration on each trial, particularly in the semantic and combined flicker conditions. Thus, in line with our original research purpose, the task used in the present study was not intended to distinguish between the effects of house and face stimuli on time dilation.

Nevertheless, we followed your suggestion and, in addition to analyzing the time perception metrics (see our reply to your previous comment), conducted supplementary analyses focusing on the house-versus-face contrast in 2-Hz ITPC values across flickering conditions, as well as the relationships between ITPC and PSE/JND. In these supplementary category-based ITPC analyses, we specifically focused on the flickering conditions, because these conditions still had a higher signal-to-noise ratio for SSVEPs compared with the random phase fluctuations in the static and scramble conditions. The general trend of findings remained the same as what was reported in the main text (Fig S6). No significant difference in 2-Hz ITPC values was found across categories (i.e., main effect of category was insignificant, *F*(1, 18) = 1.93, *p* = .182, ηp² = .097) neither did it interact with conditions (F(2, 36) = 0.33, p = .724, ηp² = .018). Moreover, the correlation between 2-Hz ITPC and time perception metrics was insignificant in all conditions for both categories (Fig S7 & Fig S8). Nevertheless, we found that faces compared to houses resulted in higher right temporoparietal entrainment which is consistent with lateralization of face processing and previous SSVEP and SWIFT studies [10–13].

This supplementary analysis is briefly referenced in the manuscript

On lines 589-590:

*“… the flickering conditions (see S1 Appendix for the face- and house-category-based analysis).”*

And is included in the supplementary materials (S1 Appendix):

*“Category specific analysis*

*The primary motivation of this study was to assess how general entrainment of higher-level ventral regions, irrespective of category, affects time dilation compared with lower-level visual regions. Consequently, in the task design of this study, the same image was used to create the standard and comparison stimuli on each trial. This allowed both low-level visual and high-level semantic confounds to be controlled and canceled out in the relative judgment of duration. Thus, we did not expect to find any category differences based on the design of this study. Nevertheless, in an exploratory analysis, we sought to investigate if face versus house images have led to cataegory-specifc modulation of time perception, ITPC, and how time perception and ITPC relate.*

*Behavioral results*

*For PSE, A 2 (category)×5 (condition) repeated-measures ANOVA showed no significant main effect of category, F(1, 18) = 0.14, p = .715, ηp² = .008, confriming that houses and faces did not affect PSEs differently. As expected, there was a significant main effect of condition, F(2.52, 45.29) = 20.96, p < .001, ηp² = .538. Moreover, the category × condition interaction was not significant, F(4, 72) = 0.60, p = .665, ηp² = .032.*

*Nevetheless, we followed up the condition effect seperately for each category to assess whether a same pattern of time dilation can be observed within each category (Fig S5). For faces, the post hoc t-test with Bonferroni correction revealed that all conditions relative to the static condition resulted in significant time dilation effect (for scramble, t(18) = -5.03, p < 0.001, 95% CI [-0.623, -0.139], Cohen’s d = -1.35; for luminance flicker, t(18) = -5.20, p < 0.001, 95% CI [-0.722, -0.172], Cohen’s d = -1.58; for semantic flicker, t(18) = -4.44, p = 0.003, 95% CI [-0.608, -0.099], Cohen’s d = -1.25; for combined flicker, t(18) = -7.04, p < 0.001, 95% CI [-0.632, -0.237], Cohen’s d = -1.53). All other comparisons were insignificant (p = 1).*

*For houses, the post hoc tests revealed the same time dilation effect for all conditions relative to the static condition (for scramble, t(18) = -5.03, p < 0.001, 95% CI [-0.553, -0.123], Cohen’s d = -1.17; for luminance flicker, t(18) = -4.61, p = 0.002, 95% CI [-0.661, -0.120], Cohen’s d = -1.35; for semantic flicker, t(18) = -4.73, p = 0.002, 95% CI [-0.681, -0.131], Cohen’s d = -1.41; for combined flicker, t(18) = -5.33, p < 0.001, 95% CI [-0.638, -0.159], Cohen’s d = -1.38). All other comparisons were insignificant (p = 1).*

*For JND, A 2 (category)×5 (condition) repeated-measures ANOVA revealed no significant main effect of category, F(1, 18) = 0.03, p = .859, ηp² = .002, confirming the stumulus category did not change the temporal sensitivry across conditions (Fig S5). The main effect of condition was not significant, F(2.63, 47.25) = 2.24, p = .104, ηp² = .111. The category × condition interaction was also not significant, F(4, 72) = 0.56, p = .692, ηp² = .030.*

*Overall, the behavioral results indicated that stimulus category did not affect time perception. Moreover, the pattern of time dilation relative to the static condition was observed within each category.*

*Fig S5. PSE and JND by category. No main effect of category was found for both PSE and JND. Moreover, similar to the findings obtained with the full image set (reported in the main text), all conditions resulted in significant time dilation (PSE shift) relative to the static condition in both the face and house stimulus categories. JND was not significantly different in any of the conditions for both categories. Error bar indicates 95% confidence interval. * p<0.05, *** p<0.001.*

*ITPC results*

*To analyze category-specific differences in ITPC, we focused only on the flickering conditions, in which the signal-to-noise ratio of the 2-Hz SSVEP was highest (compared to random noise in static and scramble conditions). The topographic maps showed that faces elicited stronger right temporoparietal entrainment than houses (Fig S6a). This is consistent with lateralization of face processing and previous SSVEP and SWIFT studies [1–5]. We analyzed the 2-Hz ITPC magnitide averaged over the pre-defined ROI (marked by dimands in Fig S6a) using A 2 (category)×3 (condition) repeated-measures ANOVA (Fig S6b). Results revealed no significant main effect of category, F(1, 18) = 1.93, p = .182, ηp² = .097. As expected, there was a significant main effect of condition, F(2, 36) = 9.86, p < .001, ηp² = .354. The category × condition interaction was not significant, F(2, 36) = 0.33, p = .724, ηp² = .018. Nevetheless, we followed up the condition effect seperately for each category.*

*For faces, the post hoc t-test with bonferroni correction revealed that combined flickers resulted in significantly higher ITPC relaive to luminance (t(18) = -2.88, p = 0.03, 95% CI [-0.247, -0.01], Cohen’s d = -0.87) and semantic flickers (t(18) = -3.10, p = 0.018, 95% CI [-0.272, -0.222], Cohen’s d = -0.10). Yet, the semantic and luminance flickers resulted in statisitcally same ITPC magnitude (t(18) = 0.44, p = 1, 95% CI [-0.09, 0.127], Cohen’s d = 0.12). For houses, post hoc tests revealed that combined flickers resulted in significantly higher ITPC relaive to luminance (t(18) = -2.86, p = 0.031, 95% CI [-0.255, -0.010], Cohen’s d = -0.9). Nevetheless, combined flickers higher ITPC magnitude relative to semantic flickers did not reach significance (t(18) = -2.33, p = 0.095, 95% CI [-0.227, 0.014], Cohen’s d = -0.72). Moreover, the semantic and luminance flickers resulted in statisitcally same ITPC magnitude (t(18) = -0.57, p = 1, 95% CI [-0.145, 0.093], Cohen’s d = -0.17).*

*Overall, the 2-Hz ITPC results obtained with the full image set (Fig 4b) were also replicated within each image category (Fig S6b). Moreover, we did not find any singifincat correlation between 2-Hz ITPC and PSE or JND for face (Fig S7) and house stimuli (Fig S8).*

*Fig S6. 2-Hz ITPC by category. (a) topographic maps of 2-Hz ITPC for face stimuli, house stimuli, and the face-versus-house contrast. Face stimuli resulted in lateralized left temporoparietal entrainment compared to house stimuli. Diamond markers indicate the pre-defined ROI same as main text. (b) the ITPC magnitude averaged over the ROI was not significantly different across face and house categories. Error bar indicates 95% confidence interval.*

*Fig. S7. Correlations between PSE and probit-transformed 2-Hz ITPC values, and between JND and probit-transformed 2-Hz ITPC values, across subjects for face stimuli. No significant correlation was found in any of the conditions.*

*Fig. S8. Correlations between PSE and probit-transformed 2-Hz ITPC values, and between JND and probit-transformed 2-Hz ITPC values, across subjects for house stimuli. No significant correlation was found in any of the conditions.”*

**3. Key Behavioral Results and Interpretation
PSE results confirm robust FITD: all dynamic conditions dilate perceived duration versus static (scramble: t(18)=5.66, p<0.001; luminance: t=5.49; semantic: t=5.44; combined: t=6.78; all BF01<0.01). Critically, no main effect of condition on PSE (rmANOVA F<1, BF01=7.2), with post-hocs non-significant (minimum t=0.97, all p>0.99, BF01>2.3 supporting equivalence). High PSE correlations across conditions (r=0.72-0.85, all p<0.001) suggest a common mechanism, plausibly SWIFT-induced ripple-motion shared by all scramble-based conditions. JND elevation is selective (luminance: t=3.8, p=0.013, BF10=29; other conditions show moderate null evidence), hinting at flicker-specific precision costs without broad impairment.
In its current form, the behavioral results do not fully disentangle the proposed hierarchical account from alternative explanations related to stimulus properties and task-specific factors. These null findings are informative: they challenge predictions of semantic > low-level amplification, but the preregistered Bayesian approach substantiates them over Type II error risk. No PSE-ITPC correlations (R²=0.03, p=0.47) further decouple behavior from neural entrainment measures, aligning with prior mixed findings in the literature. Therefore, the discussion should more explicitly acknowledge that the present data constrain the proposed interpretation but do not yet provide definitive evidence for it. I suggest discussing the possibility that reported "no result" outcome may alternatively reflect design limitations/low power rather than theoretical insights.**

We are truly grateful that you brought this important qualification of our results to our attention. We fully agree with your comment and have therefore inserted a new section titled “Limitations,” in which we explicitly explain the limitations of the current study design and the need for future replications before drawing any firm conclusions.

On lines 802-834:

“*Limitations*

*A major limitation of the current study is that we could not determine whether, in the absence of motion, entrainment of ventral regions can induce time dilation. Furthermore, the current study only assessed the FITD elicited by 2-Hz modulations Thus the generalizability of our findings to other frequency ranges remains unclear. In classic SSVEP studies using simple luminance-based flickers, it has been reported that neural oscillators have selective frequency preferences (10-, 20-, 40, and 80-Hz [20]). However, to record SSVEPs from higher-level visual regions using complex stimuli, such as faces, lower stimulation frequencies are necessary (e.g., 6 Hz [32,75]). This is in agreement with poorer temporal resolution and lower temporal frequency sensitivity of these regions [32–35]. The choice of 2-Hz modulation was informed by two considerations: previous SWIFT studies showing maximal phase clustering at 2 Hz [33], thereby maximizing the neural indices, and evidence that the strongest time dilation effects are observed at slower frequencies [26]. Thus, using 2-Hz modulation provided sufficient sensitivity to distinguish any FITD induced by entrainment from effects driven by other confounding factors. Importantly, in this study, the magnitude of ITPC within the predefined ROI did not differ between luminance and semantic flicker conditions (Fig 4b). Moreover, the analysis using individual peak electrodes in the flickering conditions did not change the findings (Fig S4 in S1 Appendix).*

*Another limitation concerns the choice of stimulus set. Specifically, in this study we focused only on face and house images, as a previous fMRI study using SWIFT showed that these stimuli can elicit maximal activation in higher-level ventral regions [62]. Thus, although we found no category-specific modulation in our main behavioral and EEG results (see S1 Appendix), it remains unclear whether a different choice of images would alter the obtained results. Moreover, because of the lengthy duration of the experiment, we presented each image only once per condition-duration combination. Although this limitation did not affect the EEG results, from a methodological perspective, future studies should include multiple repetitions of each image per condition-duration combination.*

*Overall, the results of the current study are constrained by the limitations imposed by the SWIFT technique. Future developments in image-processing techniques, as well as brain stimulation studies, may provide further insights and support the generalization of these findings.*”

**Divergence from published frequency trends (e.g., Lakhanpal et al. 2020 descending supra-second pattern) should be explicitly addressed as well—could this reflect a design error (e.g., ripple artifacts overriding semantic modulations)?**

Thank you for your comment. However, we were unable to find a publication by Lakhanpal et al. 2020, neither did we understand what was intended by descending supra-second pattern. The usage of single frequencies in study of FITD has precedents (e.g., [15–17]) and only few studies have afforded to assess a large range of frequencies (e.g., [18]). Nevertheless, following your comment (under **Additional revisions recommended**). We have provided a full justification of why 2 Hz frequency was used and have explicitly mentioned that the generalizability of our findings to other frequency ranges remains limited. Moreover, we do not believe the obtained result are caused by design ‘error’. This study specifically was designed in a way that would disentangle the effects of SWIFT ripple motions by contrasting scramble condition versus flickering conditions (Fig 3). Moreover, “ripple artifacts overriding semantic modulations” statement sounds improbable as our EEG results clearly show that SWIFT sequences have resulted in lateralized right temporoparietal activation in semantic and combined flicker conditions (Fig 4a) which is absent in scramble, and luminance flicker conditions. This confirms that the semantic modulation has essentially periodically activated the targeted higher-level ventral regions [7,14,19]. Nevertheless, as it has explicitly been mentioned in the previous and edited version of the manuscript, the major limitation of the current study is that we could not find out whether, in the absence of motion, entrainment of ventral regions can induce time dilation (see our reply to your previous comment). We, therefore, are hopeful that with advances in image analysis and/or brain stimulation techniques, the generalizability of results can be tested in future studies.

**Overall, the paper fits PLOS ONE's scope well (rigorous psychophysics, open data/methods over groundbreaking novelty). Strengths include detailed stimulus recipes (e.g., RIS U-shape selection, 3 harmonics/9 decomposition levels), apparatus specifications (VIEWPixx scanning backlight mode for precise timing), and clear task rationale.**

Thank you very much for your positive view on your study. We are truly motivated by your positive evaluation.

**Additional revisions recommended:
- Report effect sizes and confidence intervals for all t-tests and ANOVAs (e.g., Cohen's d approximately 1.3 for PSE shifts versus static baseline).**

Thank you very much for this important comment. We have added effect sizes and confidence intervals for all t-tests and ANOVAs in the manuscript and supplementary materials.

**- Discussion: Temper the V5-motion conjecture with direct citations (e.g., Kanai et al. 2006 on motion-FITD links) and quantify what constitutes "steady low-level activation" physiologically.**

Thank you very much for your meticulous comment. We re-read the manuscript and linked the V5-motion conjecture with proper citations. Moreover, based on the fMRI study which used SWIFT [14], we have clarified what steady activation of low-level visual areas refers to physiologically. The steady activation indicates that V1/V2 remained visually driven but showed no significant modulation at the SWIFT tagging frequency, suggesting that their population response was insensitive to the periodic emergence of recognizable semantic content.

This definition is first clarified in the introduction on lines 138-140:

*“…… a steady activation of lower-level visual areas is expected (i.e., V1/V2 areas are driven visually, yet their population response is insensitive to periodic semantic modulation [62]).”*

And wherever possible, a brief explanation is always mentioned when “steady activation” is used. For example, on lines 558-559, 673-674, and 712-713:

*“…. steady activation (i.e., sustained population-level visual activation [62])”*

**- Justify the 2 Hz tagging frequency choice beyond maximizing SWIFT ITPC—address why this frequency was selected despite being lower than typical SSVEP ranges (8-12 Hz alpha peak) and how this might affect entrainment strength.**

We appreciate your suggestion. In classic SSVEP studies using simple luminance-based flickers, it has been reported that neural oscillators have selective frequency preferences (10-, 20-, 40, and 80-Hz [20]). Nevertheless, as noted by Norcia et al. [21] in their seminal review, “the frequency rate giving the largest SSVEP response is not tied to the range of 8–12 Hz but rather depends on the kind of stimulus property that is modulated”. Therefore, to record SSVEPs from higher-level visual regions using complex stimuli, such as faces, lower stimulation frequencies are necessary (e.g., 6 Hz [3,21]). This is in agreement with poorer temporal resolution and lower temporal frequency sensitivity of these regions [19,21–23]. The choice of 2-Hz modulation was informed by two considerations: previous SWIFT studies showing maximal phase clustering at 2 Hz [19], thereby maximizing the neural indices, and evidence that the strongest time dilation effects are observed at slower frequencies [18]. Thus, using 2-Hz modulation provided sufficient sensitivity to distinguish any FITD induced by entrainment from effects driven by other confounding factors. Importantly, in this study, the magnitude of ITPC within the predefined ROI did not differ between luminance and semantic flicker conditions (Fig 4b). Moreover, the analysis using individual peak electrodes in the flickering conditions did not change the findings (Fig S4).

We have reflected your comment in the manuscript on lines 806-820:

*“ In classic SSVEP studies using simple luminance-based flickers, it has been reported that neural oscillators have selective frequency preferences (10-, 20-, 40, and 80-Hz [20]). However, to record SSVEPs from higher-level visual regions using complex stimuli, such as faces, lower stimulation frequencies are necessary (e.g., 6 Hz [32,75]). This is in agreement with poorer temporal resolution and lower temporal frequency sensitivity of this region [32-35]. The choice of 2-Hz modulation was informed by two considerations: previous SWIFT studies showing maximal phase clustering at 2 Hz [33], thereby maximizing the neural indices, and evidence that the strongest time dilation effects are observed at slower frequencies [26]. Thus, using 2-Hz modulation provided sufficient sensitivity to distinguish any FITD induced by entrainment from effects driven by other confounding factors. Importantly, in this study, the magnitude of ITPC within the predefined ROI did not differ between luminance and semantic flicker conditions (Fig 4b). Moreover, the analysis using individual peak electrodes in the flickering conditions did not change the findings (Fig S4 in S1 Appendix).”*

**- Figures: Add error bars (SEM or 95% CI) to PSE/JND bar plots; clarify which electrodes constitute the ROI in ITPC topography maps.**

Thank you very much for your comment. All PSE/JND bar plots in Fig 6 are already accompanied by proper 95% CI error bars as is evident in the figure and the caption. However, per your suggestion, we have also marked the ROI in ITPC maps in Fig 4a.

**- Language: Fix minor typos (e.g., "rational" → "rationale" line 177; "stimulis" → "stimuli" throughout); ensure consistent abbreviation usage (FITD, SSVEP).**

Thank you very much for your comment. We have fixed the typos and have replaced/ rephrased all the incorrect “stimuli’s” phrase throughout of the manuscript. We have re-read the manuscript to ensure no additional typos have been left unnoticed.

**References**

1.	Cohen MX. Analyzing Neural Time Series Data. Analyzing Neural Time Series Data. The MIT Press; 2014. doi:10.7551/mitpress/9609.001.0001

2.	Benjamini Y, Hochberg Y. Controlling the False Discovery Rate: A Practical and Powerful Approach to Multiple Testing. J R Stat Soc Ser B Stat Methodol. 1995;57: 289–300. doi:10.1111/j.2517-6161.1995.tb02031.x

3.	Alonso-Prieto E, Belle G Van, Liu-Shuang J, Norcia AM, Rossion B. The 6 Hz fundamental stimulation frequency rate for individual face discrimination in the right occipito-temporal cortex. Neuropsychologia. 2013;51: 2863–75. doi:10.1016/j.neuropsychologia.2013.08.018

4.	Bentin S, Sagiv N, Mecklinger A, Friederici A, von Cramon YD. Priming visual face-processing mechanisms: electrophysiological evidence. Psychol Sci. 2002;13: 190–3. doi:10.1111/1467-9280.00435

5.	Rossion B. Understanding face perception by means of human electrophysiology. Trends Cogn Sci. 2014;18: 310–8. doi:10.1016/j.tics.2014.02.013

6.	Lueschow A, Sander T, Boehm SG, Nolte G, Trahms L, Curio G. Looking for faces: Attention modulates early occipitotemporal object processing. Psychophysiology. 2004;41: 350–60. doi:10.1111/j.1469-8986.2004.00159.x

7.	Gagsch F, Valuch C, Albrecht T. Measuring attentional selection of object categories using hierarchical frequency tagging. J Vis. 2024;24: 8. doi:10.1167/jov.24.7.8

8.	Martin JR, Sackur J, Anlló H, Naish P, Dienes Z. Perceiving time differences when you should not: Applying the El Greco fallacy to hypnotic time distortions. Front Psychol. 2016;7: 1–8. doi:10.3389/fpsyg.2016.01309

9.	Firestone C, Scholl BJ. “Top-Down” Effects Where None Should Be Found: The El Greco Fallacy in Perception Research. Psychol Sci. 2014;25: 38–46. doi:10.1177/0956797613485092

10.	Vettori S, Dzhelyova M, Van der Donck S, Jacques C, Steyaert J, Rossion B, et al. Frequency-Tagging Electroencephalography of Superimposed Social and Non-Social Visual Stimulation Streams Reveals Reduced Saliency of Faces in Autism Spectrum Disorder. Front Psychiatry. 2020;11: 1–12. doi:10.3389/fpsyt.2020.00332

11.	Wardle SG, Brendan Ritchie J. Can object category-selectivity in the ventral visual pathway be explained by sensitivity to low-level image properties? J Neurosci. 2014;34: 14817–14819. doi:10.1523/JNEUROSCI.3566-14.2014

12.	Andrews TJ, Watson DM, Rice GE, Hartley T. Low-level properties of natural images predict topographic patterns of neural response in the ventral visual pathway. J Vis. 2015;15: 1–12. doi:10.1167/15.7.3

13.	Willenbockel V, Sadr J, Fiset D, Horne GO, Gosselin F, Tanaka JW. Controlling low-level image properties: The SHINE toolbox. Behav Res Methods. 2010;42: 671–684. doi:10.3758/BRM.42.3.671

14.	Koenig-Robert R, VanRullen R, Tsuchiya N. Semantic Wavelet-Induced Frequency-Tagging (SWIFT) Periodically Activates Category Selective Areas While Steadily Activating Early Visual Areas. PLoS One. 2015;10: e0144858. doi:10.1371/journal.pone.0144858

15.	Hashimoto Y, Yotsumoto Y. Effect of Temporal Frequency Spectra of Flicker on Time Perception: Behavioral Testing and Simulations Using a Striatal Beat Frequency Model. Timing Time Percept. 2015;3: 201–222. doi:10.1163/22134468-03002049

16.	Li L, Ito S, Yotsumoto Y. Effect of change saliency and neural entrainment on flicker-induced time dilation. J Vis. 2020;20: 15. doi:10.1167/jov.20.6.15

17.	Horr NK, Wimber M, Di Luca M. Perceived time and temporal structure: Neural entrainment to isochronous stimulation increases duration estimates. Neuroimage. 2016;132: 148–156. doi:10.1016/j.neuroimage.2016.02.011

18.	Herbst SK, Javadi AH, van der Meer E, Busch NA. How Long Depends on How Fast-Perceived Flicker Dilates Subjective Duration. PLoS One. 2013;8. doi:10.1371/journal.pone.0076074

19.	Koenig-Robert R, VanRullen R. SWIFT: a novel method to track the neural correlates of recognition. Neuroimage. 2013;81: 273–282. doi:10.1016/j.neuroimage.2013.04.116

20.	Herrmann CS. Human EEG responses to 1-100 Hz flicker: resonance phenomena in visual cortex and their potential correlation to cognitive phenomena. Exp brain Res. 2001;137: 346–53. doi:10.1007/s002210100682

21.	Norcia AM, Appelbaum LG, Ales JM, Cottereau BR, Rossion B. The steady-state visual evoked potential in vision research: A review. J Vis. 2015;15: 4. doi:10.1167/15.6.4

22.	Holcombe AO. Seeing slow and seeing fast: two limits on perception. Trends Cogn Sci. 2009;13: 216–21. doi:10.1016/j.tics.2009.02.005

23.	Clifford CWG, Holcombe AO, Pearson J. Rapid global form binding with loss of associated colors. J Vis. 2004;4: 1090–101. doi:10.1167/4.12.8
