# Supplementary material for: Toward understanding the role of visual hierarchy in flicker-induced time dilation: A preregistered study
Source: PLoS One. 2026 Oct 13;21(10):e0337307. doi: 10.1371/journal.pone.0337307 (PMC13660902; doi:10.1371/journal.pone.0337307)
Supplement: Supplementary file 3 [file pone.0337307.s004.docx]

We wish to express our appreciation to the editor and the reviewers for their meticulous reading of our manuscript and the insightful comments we have received which have helped us significantly improve our manuscript.

Next, we offer detailed responses to the editor and reviewers’ comments. The editor and reviewers’ comments are shown in BOLD font, our responses are in blue, and the sentences in the revised manuscript are shown in italic. All the changes to the manuscript are highlighted by the yellow color.

**Dear Dr. Houshmand Chatroudi,

Thank you for submitting your manuscript to PLOS One. After careful consideration, we feel that it has merit but does not fully meet PLOS One’s publication criteria as it currently stands. Therefore, we invite you to submit a revised version of the manuscript that addresses the points raised during the review process.**

**As you will note, one of the original Reviewers appreciated the changes made and requested only one further clarification regarding the filtering process. I have carefully reviewed the manuscript and verified that you have satisfactorily addressed also the comments of the other original Reviewer, who was unable to evaluate the new version of the work. I am therefore pleased to inform you that the only requirement is to add the requested details regarding the filters used.**

We sincerely thank the editor for carefully reviewing our revised manuscript and assessing our responses to both reviewers. We have now added the requested details regarding the low- and high-pass filters, including their transition bandwidths and phase response. The revised text is provided below in our response to the Reviewer’s comment.

**Please include the following items when submitting your revised manuscript:**

- **A letter that responds to each point raised by the academic editor and reviewer(s). You should upload this letter as a separate file labeled 'Response to Reviewers'.**
- **A marked-up copy of your manuscript that highlights changes made to the original version. You should upload this as a separate file labeled 'Revised Manuscript with Track Changes'.**
- **An unmarked version of your revised paper without tracked changes. You should upload this as a separate file labeled 'Manuscript'.**

**If you would like to make changes to your financial disclosure, please include your updated statement in your cover letter. Guidelines for resubmitting your figure files are available below the reviewer comments at the end of this letter.**

Thank you for raising these points. We have acted accordingly and submitted the requested files. We do not wish to change the financial disclosure information, and we have ensured our images meet the requirements set by PLOS One. **If applicable, we recommend that you deposit your laboratory protocols in protocols.io to enhance the reproducibility of your results. Protocols.io assigns your protocol its own identifier (DOI) so that it can be cited independently in the future. For instructions see:** [**https://journals.plos.org/plosone/s/submission-guidelines#loc-laboratory-protocols**](https://journals.plos.org/plosone/s/submission-guidelines#loc-laboratory-protocols)**. Additionally, PLOS One offers an option for publishing peer-reviewed Lab Protocol articles, which describe protocols hosted on protocols.io. Read more information on sharing protocols at** [**https://plos.org/protocols?utm_medium=editorial-email&utm_source=authorletters&utm_campaign=protocols**](https://plos.org/protocols?utm_medium=editorial-email&utm_source=authorletters&utm_campaign=protocols)**.**

**As the corresponding author, your ORCID iD is verified in the submission system and will appear in the published article. PLOS supports the use of ORCID, and we encourage all coauthors to register for an ORCID iD and use it as well. Please encourage your coauthors to verify their ORCID iD within the submission system before final acceptance, as unverified ORCID iDs will not appear in the published article. *Only* the individual author can complete the verification step; PLOS staff *cannot* verify ORCID iDs on behalf of authors.

We look forward to receiving your revised manuscript.**

**Kind regards,**

**Giulia Prete
Academic Editor
PLOS One**

**Journal Requirements:**

**1. If the reviewer comments include a recommendation to cite specific previously published works, please review and evaluate these publications to determine whether they are relevant and should be cited. There is no requirement to cite these works unless the editor has indicated otherwise. 

2. Please review your reference list to ensure that it is complete and correct. If you have cited papers that have been retracted, please include the rationale for doing so in the manuscript text, or remove these references and replace them with relevant current references. Any changes to the reference list should be mentioned in the rebuttal letter that accompanies your revised manuscript. If you need to cite a retracted article, indicate the article’s retracted status in the References list and also include a citation and full reference for the retraction notice.**

Thank you for this comment. We have re-read and ensured the references are correct and accurate. **Reviewers' comments:**

**6. Review Comments to the Author

Please use the space provided to explain your answers to the questions above. You may also include additional comments for the author, including concerns about dual publication, research ethics, or publication ethics. (Please upload your review as an attachment if it exceeds 20,000 characters)**

**Reviewer #1: Thank you for addressing all previous comments thoroughly and providing additional analyses, which strengthen the robustness of the manuscript.**

We sincerely appreciate your positive evaluation of our manuscript and are pleased that our revisions have addressed your insightful comments. **I just have one minor point: there was a small misunderstanding of what was meant by filters in my previous comment. While I appreciate the description of the wavelets, please also describe the details of the low- and high-pass filters (l.361, 362: transition bandwidth, phase response).**

Thank you for clarifying this point, and we apologize for misunderstanding the previous comment. We have revised the preprocessing description to specify the filter design, orders, transition bandwidths, and phase response. The low-pass and high-pass filters were Hamming-windowed sinc FIR filters with transition bandwidths of 7.5 Hz and 0.5 Hz, respectively (using EEGLAB’s *pop_eegfiltnew* function). Both were applied with group-delay compensation, yielding zero-phase, noncausal filtering.

This description is now added on lines 348-353:

“*EEG data were first low-pass filtered at 30 Hz and subsequently, high-pass filtered at 0.5 Hz using Hamming-windowed sinc finite impulse response (FIR) filters implemented in EEGLAB (version 2024.2 [67]; pop_eegfiltnew). The low-pass and high-pass filters had transition bandwidths of 7.5 Hz and 0.5 Hz and automatically determined orders of 440 and 6600, respectively. Both filters were linear-phase and applied with group-delay compensation, yielding zero-phase, noncausal filtering.*”

**To ensure your figures meet our technical requirements, please review our figure guidelines: https://journals.plos.org/plosone/s/figures**

**You may also use PLOS’s free figure tool, NAAS, to help you prepare publication quality figures: https://journals.plos.org/plosone/s/figures#loc-tools-for-figure-preparation.**

**NAAS will assess whether your figures meet our technical requirements by comparing each figure against our figure specifications.**

 Thank you very much for this point. The figures we submitted are all checked by NAAS and have passed the quality check in accordance with the journal guidelines.
