# Supplementary material for: Toward understanding the role of visual hierarchy in flicker-induced time dilation: A preregistered study
Source: PLoS One. 2026 Oct 13;21(10):e0337307. doi: 10.1371/journal.pone.0337307 (PMC13660902; doi:10.1371/journal.pone.0337307)
Supplement: Peer review history

# Peer review: Decision Letter 0

**DOI:** 10.1371/journal.pone.0337307.r001

**Authors:** Giulia Prete

23 Mar 2026

Dear Dr. Houshmand Chatroudi,

Thank you for submitting your manuscript to PLOS ONE. After careful consideration, we feel that it has merit but does not fully meet PLOS ONE’s publication criteria as it currently stands. Therefore, we invite you to submit a revised version of the manuscript that addresses the points raised during the review process.

Please submit your revised manuscript by May 07 2026 11:59PM. If you will need more time than this to complete your revisions, please reply to this message or contact the journal office at plosone@plos.org. When you’re ready to submit your revision, log on to https://www.editorialmanager.com/pone/ and select the 'Submissions Needing Revision' folder to locate your manuscript file.

- A letter that responds to each point raised by the academic editor and reviewer(s). You should upload this letter as a separate file labeled 'Response to Reviewers'.
- A marked-up copy of your manuscript that highlights changes made to the original version. You should upload this as a separate file labeled 'Revised Manuscript with Track Changes'.
- An unmarked version of your revised paper without tracked changes. You should upload this as a separate file labeled 'Manuscript'.

If you would like to make changes to your financial disclosure, please include your updated statement in your cover letter. Guidelines for resubmitting your figure files are available below the reviewer comments at the end of this letter.

If applicable, we recommend that you deposit your laboratory protocols in protocols.io to enhance the reproducibility of your results. Protocols.io assigns your protocol its own identifier (DOI) so that it can be cited independently in the future. For instructions see: https://journals.plos.org/plosone/s/submission-guidelines#loc-laboratory-protocols. Additionally, PLOS ONE offers an option for publishing peer-reviewed Lab Protocol articles, which describe protocols hosted on protocols.io. Read more information on sharing protocols at https://plos.org/protocols?utm_medium=editorial-email&utm_source=authorletters&utm_campaign=protocols.

We look forward to receiving your revised manuscript.

Kind regards,

Giulia Prete

Academic Editor

PLOS One

Journal Requirements:

When submitting your revision, we need you to address these additional requirements.

1. Please ensure that your manuscript meets PLOS ONE's style requirements, including those for file naming. The PLOS ONE style templates can be found at

https://journals.plos.org/plosone/s/file?id=wjVg/PLOSOne_formatting_sample_main_body.pdf and

https://journals.plos.org/plosone/s/file?id=ba62/PLOSOne_formatting_sample_title_authors_affiliations.pdf

2. We note that you have included the phrase “data not shown” in your manuscript. Unfortunately, this does not meet our data sharing requirements. PLOS does not permit references to inaccessible data. We require that authors provide all relevant data within the paper, Supporting Information files, or in an acceptable, public repository. Please add a citation to support this phrase or upload the data that corresponds with these findings to a stable repository (such as Figshare or Dryad) and provide and URLs, DOIs, or accession numbers that may be used to access these data. Or, if the data are not a core part of the research being presented in your study, we ask that you remove the phrase that refers to these data.

3. We note that Figures 1, 2 and Movie S1 in Supporting information file includes an image of a participant  in the study.

As per the PLOS ONE policy (http://journals.plos.org/plosone/s/submission-guidelines#loc-human-subjects-research) on papers that include identifying, or potentially identifying, information, the individual(s) or parent(s)/guardian(s) must be informed of the terms of the PLOS open-access (CC-BY) license and provide specific permission for publication of these details under the terms of this license. Please download the Consent Form for Publication in a PLOS Journal (http://journals.plos.org/plosone/s/file?id=8ce6/plos-consent-form-english.pdf). The signed consent form should not be submitted with the manuscript, but should be securely filed in the individual's case notes. Please amend the methods section and ethics statement of the manuscript to explicitly state that the patient/participant has provided consent for publication: “The individual in this manuscript has given written informed consent (as outlined in PLOS consent form) to publish these case details”.

If you are unable to obtain consent from the subject of the photograph, you will need to remove the figure and any other textual identifying information or case descriptions for this individual.

4. Please include captions for your Supporting Information files at the end of your manuscript, and update any in-text citations to match accordingly. Please see our Supporting Information guidelines for more information: http://journals.plos.org/plosone/s/supporting-information.

5. If the reviewer comments include a recommendation to cite specific previously published works, please review and evaluate these publications to determine whether they are relevant and should be cited. There is no requirement to cite these works unless the editor has indicated otherwise.

[Note: HTML markup is below. Please do not edit.]

Reviewers' comments:

Reviewer's Responses to Questions

**Comments to the Author**

1. Is the manuscript technically sound, and do the data support the conclusions?

Reviewer #1: Yes

Reviewer #2: Yes

**********

2. Has the statistical analysis been performed appropriately and rigorously?

Reviewer #1: Yes

Reviewer #2: Yes

**********

3. Have the authors made all data underlying the findings in their manuscript fully available?

The PLOS Data policy

Reviewer #1: Yes

Reviewer #2: Yes

**********

4. Is the manuscript presented in an intelligible fashion and written in standard English?

Reviewer #1: Yes

Reviewer #2: Yes

**********

Reviewer #1: This pre-registered study tests whether subjective time dilation induced by visual flicker results from neural entrainment in low or high level visual area. The design cleverly combines basic luminance and semantic flicker (same low-level image properties, but periodic occurrence of a face or house) in a forced choice interval duration comparison task.

The EEG data show robust phase clustering by the flickering stimuli both for luminance and semantic flickers, hence clear evidence for phase entrainment.

The behavioral data show no specific time dilation with respect to the flicker, as all conditions, including the scrambled one are dilated compared to the static one. A small difference is observed in JND, but neither the PSD nor the JND effects correlate with the phase entrainment quantified by the ITC.

The study design and analyses are very clear. Despite the null effect between entrainment and duration dilation, this work is a valuable addition to the literature; its methodological rigor provides strong evidence for the lack of a relationship. I also found the discussion particularly compelling, offering insightful arguments to contextualize the results.

Regarding the methodology, the ITPC analysis was quite broad (using the same electrodes for all participants, averaged over a fixed window). Would extracting ITPC from individual peak electrodes, and perhaps even per condition, allow for a more refined assessment of entrainment and its potential links to timing behavior?

To clarify:

Where the flicker conditions all intermixed on a trial by trial basis?

Report details of EEG filters, which are especially relevant for the phase analysis.

EEG epochs: with respect to which events were the epochs created? Only for the comparison stimuli?

Was an extra baseline correction applied to the time-frequency transforms or ITPC values?

p.17, l. 379-380: what is meant by 'time window region of interest'?

Explain why the probit transform of ITPC values was performed in the main manuscript so the reader does not have to go back and forth between the preregistration and the paper.

p. 20, l.414 – 422: This could be formulated more clearly. Does the analysis check for main effects of condition and ITPC on behavior (DV)?

Minor:

p.6, l.112: 'The ventral stream of the vision' -> in vision

p. 9, l.182 using histogram equalization function -> 'a histogram'..

p. 25, l. 566 Bayse factor -> Bayes factor (also occurs at least once in the Discussion)

Figure resolution appears low, but might be due to automatic pdf conversion?

Reviewer #2: This manuscript reports a preregistered experiment examining the cortical underpinnings of flicker-induced time dilation (FITD), specifically whether higher-level ventral visual stream regions contribute via selective neural entrainment using semantic wavelet-induced frequency tagging (SWIFT). The study integrates sophisticated stimulus generation with a robust psychophysical duration comparison task, yielding clear evidence of time dilation across dynamic conditions relative to static baselines, while finding no predicted modulation by flicker hierarchy. Although the preregistration and open science practices are commendable, the manuscript would benefit from a more cautious interpretation of the null and near-null findings. The work aligns well with PLOS ONE's emphasis on rigorous, transparent methodology in perception research.

1. Strengths in Preregistration and Open Science Practices

The authors adhered closely to their detailed preregistration, which specifies hypotheses, experimental conditions (static, scramble, luminance flicker, semantic flicker, combined flicker at 2 Hz tagging frequency), sample size (N=21 targeted, N=20 after preregistered exclusion for poor temporal sensitivity), analysis pipeline (Palamedes psychometric fits with 2% lapse rates, repeated-measures ANOVA on PSE/JND, Bayes factors via JASP, ITPC correlations), and exclusion criteria. This fidelity reduces concerns about flexibility in data analysis and enhances credibility. Exploratory analyses (e.g., harmonics, power spectra, ERPs) are transparently flagged as such, avoiding HARKing. Raw data, scripts, and supplementary materials are publicly available on OSF, including stimulus movies—exemplary for reproducibility. The power analysis (targeting d=0.8 for ANOVA) is justified and reported, with post-hoc sensitivity noted.

2. Behavioral Methods and Task Design

The core behavioral paradigm—a 2-interval forced-choice (2IFC) duration discrimination task—is methodologically strong. Standard stimuli (2.5s fixed duration) in each of the five conditions are precisely matched to their static comparison stimuli (1-4s in 0.5s steps, same source image), effectively controlling for low-level (luminance, contrast, pixel modulation spectra) and high-level (semantic content: faces/houses) confounds between intervals. This within-trial matching is a key strength, isolating flicker manipulations from adaptation or image-specific biases. SWIFT normalization (CLAHE contrast, pixel FFT flattening to 7.2 Hz cutoff, frame-wise luminance equating) rigorously equates non-target features across semantic/scramble conditions, while in-phase luminance overlay in combined flickers tests hierarchical tagging as intended.

Trial structure minimizes confounds since randomized fixation/blank/ISI durations prevent counting. Moreover, instructions emphasize no counting and eye fixation. Psychometric fits yield PSE (bias) and JND (precision) as intuitive metrics for FITD magnitude and temporal sensitivity cost.

As for blocks structure, on one hand single repetition per image-duration-condition (560 trials over 7 blocks with rests) prevents excessive participants' fatigue. On the other hand, a potential flaw may arise in such low repetitions (yielding low power): with 16 images and only 1 repetition per image-duration-condition combination, image-specific noise is uncontrolled. Moreover, faces versus houses were selected based on fMRI motivation for ventral activation, yet no category analysis is reported despite the ventral stream hypothesis.

3. Key Behavioral Results and Interpretation

PSE results confirm robust FITD: all dynamic conditions dilate perceived duration versus static (scramble: t(18)=5.66, p<0.001; luminance: t=5.49; semantic: t=5.44; combined: t=6.78; all BF01<0.01). Critically, no main effect of condition on PSE (rmANOVA F<1, BF01=7.2), with post-hocs non-significant (minimum t=0.97, all p>0.99, BF01>2.3 supporting equivalence). High PSE correlations across conditions (r=0.72-0.85, all p<0.001) suggest a common mechanism, plausibly SWIFT-induced ripple-motion shared by all scramble-based conditions. JND elevation is selective (luminance: t=3.8, p=0.013, BF10=29; other conditions show moderate null evidence), hinting at flicker-specific precision costs without broad impairment.

In its current form, the behavioral results do not fully disentangle the proposed hierarchical account from alternative explanations related to stimulus properties and task-specific factors. These null findings are informative: they challenge predictions of semantic > low-level amplification, but the preregistered Bayesian approach substantiates them over Type II error risk. No PSE-ITPC correlations (R²=0.03, p=0.47) further decouple behavior from neural entrainment measures, aligning with prior mixed findings in the literature.

Therefore, the discussion should more explicitly acknowledge that the present data constrain the proposed interpretation but do not yet provide definitive evidence for it. I suggest discussing the possibility that reported "no result" outcome may alternatively reflect design limitations/low power rather than theoretical insights. Divergence from published frequency trends (e.g., Lakhanpal et al. 2020 descending supra-second pattern) should be explicitly addressed as well—could this reflect a design error (e.g., ripple artifacts overriding semantic modulations)?

Overall, the paper fits PLOS ONE's scope well (rigorous psychophysics, open data/methods over groundbreaking novelty). Strengths include detailed stimulus recipes (e.g., RIS U-shape selection, 3 harmonics/9 decomposition levels), apparatus specifications (VIEWPixx scanning backlight mode for precise timing), and clear task rationale.

Additional revisions recommended:

- Report effect sizes and confidence intervals for all t-tests and ANOVAs (e.g., Cohen's d approximately 1.3 for PSE shifts versus static baseline).

- Discussion: Temper the V5-motion conjecture with direct citations (e.g., Kanai et al. 2006 on motion-FITD links) and quantify what constitutes "steady low-level activation" physiologically.

- Justify the 2 Hz tagging frequency choice beyond maximizing SWIFT ITPC—address why this frequency was selected despite being lower than typical SSVEP ranges (8-12 Hz alpha peak) and how this might affect entrainment strength.

- Figures: Add error bars (SEM or 95% CI) to PSE/JND bar plots; clarify which electrodes constitute the ROI in ITPC topography maps.

- Language: Fix minor typos (e.g., "rational" → "rationale" line 177; "stimulis" → "stimuli" throughout); ensure consistent abbreviation usage (FITD, SSVEP).

**********

what does this mean?). If published, this will include your full peer review and any attached files.

If you choose “no”, your identity will remain anonymous but your review may still be made public.

**Do you want your identity to be public for this peer review?**  For information about this choice, including consent withdrawal, please see our Privacy Policy

Reviewer #1: **Yes:**  Sophie Herbst

Reviewer #2: No

**********

[NOTE: If reviewer comments were submitted as an attachment file, they will be attached to this email and accessible via the submission site. Please log into your account, locate the manuscript record, and check for the action link "View Attachments". If this link does not appear, there are no attachment files.]

To ensure your figures meet our technical requirements, please review our figure guidelines: https://journals.plos.org/plosone/s/figures

You may also use PLOS’s free figure tool, NAAS, to help you prepare publication quality figures: https://journals.plos.org/plosone/s/figures#loc-tools-for-figure-preparation.

NAAS will assess whether your figures meet our technical requirements by comparing each figure against our figure specifications.

# Author comment: Author response to Decision Letter 1

**DOI:** 10.1371/journal.pone.0337307.r002

14 Jul 2026

Dear Editor and Reviewers,

Thank you very much for reading our manuscript and providing insightful comments.

Our reply to the comments is provided in the file titled 'Response to Reviewers.docx'

Best regards,

Amirmahmoud Houshmand Chatroudi,

Post-doctoral researcher

the University of Tokyo, Japan

email: bellhoushmand@gmail.com

Attachment Submitted filename: Response to Reviewers.docx

# Peer review: Decision Letter 1

**DOI:** 10.1371/journal.pone.0337307.r003

**Authors:** Giulia Prete

18 Sep 2026

Dear Dr. Houshmand Chatroudi,

Thank you for submitting your manuscript to PLOS One. After careful consideration, we feel that it has merit but does not fully meet PLOS One’s publication criteria as it currently stands. Therefore, we invite you to submit a revised version of the manuscript that addresses the points raised during the review process.

Please submit your revised manuscript by Nov 02 2026 11:59PM. If you will need more time than this to complete your revisions, please reply to this message or contact the journal office at plosone@plos.org. When you're ready to submit your revision, log on to https://www.editorialmanager.com/pone/ and select the 'Submissions Needing Revision' folder to locate your manuscript file.

- A letter that responds to each point raised by the academic editor and reviewer(s). You should upload this letter as a separate file labeled 'Response to Reviewers'.
- A marked-up copy of your manuscript that highlights changes made to the original version. You should upload this as a separate file labeled 'Revised Manuscript with Track Changes'.
- An unmarked version of your revised paper without tracked changes. You should upload this as a separate file labeled 'Manuscript'.

If you would like to make changes to your financial disclosure, please include your updated statement in your cover letter. Guidelines for resubmitting your figure files are available below the reviewer comments at the end of this letter.

If applicable, we recommend that you deposit your laboratory protocols in protocols.io to enhance the reproducibility of your results. Protocols.io assigns your protocol its own identifier (DOI) so that it can be cited independently in the future. For instructions see: https://journals.plos.org/plosone/s/submission-guidelines#loc-laboratory-protocols. Additionally, PLOS One offers an option for publishing peer-reviewed Lab Protocol articles, which describe protocols hosted on protocols.io. Read more information on sharing protocols at https://plos.org/protocols?utm_medium=editorial-email&utm_source=authorletters&utm_campaign=protocols.

As the corresponding author, your ORCID iD is verified in the submission system and will appear in the published article. PLOS supports the use of ORCID, and we encourage all coauthors to register for an ORCID iD and use it as well. Please encourage your coauthors to verify their ORCID iD within the submission system before final acceptance, as unverified ORCID iDs will not appear in the published article. *Only*  the individual author can complete the verification step; PLOS staff *cannot*  verify ORCID iDs on behalf of authors.

We look forward to receiving your revised manuscript.

Kind regards,

Giulia Prete

Academic Editor

PLOS One

**Journal Requirements:**

1. If the reviewer comments include a recommendation to cite specific previously published works, please review and evaluate these publications to determine whether they are relevant and should be cited. There is no requirement to cite these works unless the editor has indicated otherwise.

2. Please review your reference list to ensure that it is complete and correct. If you have cited papers that have been retracted, please include the rationale for doing so in the manuscript text, or remove these references and replace them with relevant current references. Any changes to the reference list should be mentioned in the rebuttal letter that accompanies your revised manuscript. If you need to cite a retracted article, indicate the article’s retracted status in the References list and also include a citation and full reference for the retraction notice.

[Note: HTML markup is below. Please do not edit.]

Reviewers' comments:

Reviewer's Responses to Questions

**Comments to the Author**

Reviewer #1: All comments have been addressed

**********

2. Is the manuscript technically sound, and do the data support the conclusions?

Reviewer #1: Yes

**********

3. Has the statistical analysis been performed appropriately and rigorously?

Reviewer #1: Yes

**********

4. Have the authors made all data underlying the findings in their manuscript fully available?

The PLOS Data policy

Reviewer #1: Yes

**********

5. Is the manuscript presented in an intelligible fashion and written in standard English?

Reviewer #1: Yes

**********

Reviewer #1: Thank you for addressing all previous comments thoroughly and providing additional analyses, which strengthen the robustness of the manuscript.

I just have one minor point: there was a small misunderstanding of what was meant by filters in my previous comment. While I appreciate the description of the wavelets, please also describe the details of the low- and high-pass filters (l.361, 362: transition bandwidth, phase response).

**********

what does this mean?). If published, this will include your full peer review and any attached files.

If you choose “no”, your identity will remain anonymous but your review may still be made public.

**Do you want your identity to be public for this peer review?**  For information about this choice, including consent withdrawal, please see our Privacy Policy

Reviewer #1: No

**********

[NOTE: If reviewer comments were submitted as an attachment file, they will be attached to this email and accessible via the submission site. Please log into your account, locate the manuscript record, and check for the action link "View Attachments". If this link does not appear, there are no attachment files.]

To ensure your figures meet our technical requirements, please review our figure guidelines: https://journals.plos.org/plosone/s/figures

You may also use PLOS’s free figure tool, NAAS, to help you prepare publication quality figures: https://journals.plos.org/plosone/s/figures#loc-tools-for-figure-preparation.

NAAS will assess whether your figures meet our technical requirements by comparing each figure against our figure specifications.

# Author comment: Author response to Decision Letter 2

**DOI:** 10.1371/journal.pone.0337307.r004

23 Sep 2026

Please find our response to the reviewers in the "Response to Reviewers.docx" file.

Attachment Submitted filename: Response_to_Reviewers_auresp_2.docx

# Editor report: Decision Letter 2

**DOI:** 10.1371/journal.pone.0337307.r005

**Authors:** Giulia Prete

27 Sep 2026

Toward Understanding the Role of Visual Hierarchy in Flicker-induced Time Dilation: A Preregistered Study

PONE-D-25-59596R2

Dear Dr. Houshmand Chatroudi,

We’re pleased to inform you that your manuscript has been judged scientifically suitable for publication and will be formally accepted for publication once it meets all outstanding technical requirements.

Within one week, you’ll receive an e-mail detailing the required amendments. When these have been addressed, you’ll receive a formal acceptance letter and your manuscript will be scheduled for publication.

An invoice will be generated when your article is formally accepted. Please note, if your institution has a publishing partnership with PLOS and your article meets the relevant criteria, all or part of your publication costs will be covered. Please make sure your user information is up-to-date by logging into Editorial Manager at Editorial Manager® and clicking the ‘Update My Information' link at the top of the page. For questions related to billing, please contact billing support.

If your institution or institutions have a press office, please notify them about your upcoming paper to help maximize its impact. If they’ll be preparing press materials, please inform our press team as soon as possible -- no later than 48 hours after receiving the formal acceptance. Your manuscript will remain under strict press embargo until 2 pm Eastern Time on the date of publication. For more information, please contact onepress@plos.org.

Kind regards,

Giulia Prete

Academic Editor

PLOS One

# Editor report: Acceptance letter

**DOI:** 10.1371/journal.pone.0337307.r006

**Authors:** Giulia Prete

PONE-D-25-59596R2

PLOS One

Dear Dr. Houshmand Chatroudi,

I'm pleased to inform you that your manuscript has been deemed suitable for publication in PLOS One. Congratulations! Your manuscript is now being handed over to our production team.

At this stage, our production department will prepare your paper for publication. This includes ensuring the following:

* All references, tables, and figures are properly cited

* All relevant supporting information is included in the manuscript submission,

* There are no issues that prevent the paper from being properly typeset

You will receive further instructions from the production team, including instructions on how to review your proof when it is ready. Please keep in mind that we are working through a large volume of accepted articles, so please give us a few days to review your paper and let you know the next and final steps.

Lastly, if your institution or institutions have a press office, please let them know about your upcoming paper now to help maximize its impact. If they'll be preparing press materials, please inform our press team within the next 48 hours. Your manuscript will remain under strict press embargo until 2 pm Eastern Time on the date of publication. For more information, please contact onepress@plos.org.

You will receive an invoice from PLOS for your publication fee after your manuscript has reached the completed accept phase. If you receive an email requesting payment before acceptance or for any other service, this may be a phishing scheme. Learn how to identify phishing emails and protect your accounts at https://explore.plos.org/phishing.

If we can help with anything else, please email us at customercare@plos.org.

Thank you for submitting your work to PLOS One and supporting open access.

Kind regards,

PLOS One Editorial Office Staff

on behalf of

Dr. Giulia Prete

Academic Editor

PLOS One
